# Practicing Interoceptive Sensitivity as a Couple: A Mixed-Methods Acceptance Analysis of a Dyadic vs. Single Pilot Randomized Controlled Trial

**DOI:** 10.3390/nu16121949

**Published:** 2024-06-19

**Authors:** Nadja-R. Baer, Noemi Vanessa Grissmer, Liane Schenk, Hanna R. Wortmann, Petra Warschburger, Ulrike A. Gisch

**Affiliations:** 1 NutriAct—Competence Cluster Nutrition Research Berlin-Potsdam, 14558 Nuthetal, Germany; noemi-vanessa.grissmer@charite.de (N.V.G.); liane.schenk@charite.de (L.S.); hwortman@uni-potsdam.de (H.R.W.); petra.warschburger@uni-potsdam.de (P.W.); ulrike.gisch@ernaehrung.uni-giessen.de (U.A.G.); 2 Institute for Medical Sociology and Rehabilitation Science, Charité—Universitätsmedizin Berlin, Charitéplatz 1, 10117 Berlin, Germany; 3 Counseling Psychology, Department of Psychology, University of Potsdam, Karl-Liebknecht-Str. 24-25, 14476 Potsdam, Germany; 4 Department of Nutritional Psychology, Institute of Nutritional Science, Justus Liebig University Giessen, Senckenbergstr. 3, 35390 Giessen, Germany

**Keywords:** intuitive eating, interoceptive sensitivity, healthy eating practice, interoception-based training, dyadic intervention, couple influence, acceptance, Theoretical Framework of Acceptability (TFA), mixed methods, Pillar Integration Process (PIP)

## Abstract

Training interoceptive sensitivity (IS) might be a first step in effectively promoting intuitive eating (IE). A dyadic interoception-based pilot randomized controlled trial was conducted to increase IE among couples aged 50+. The training consisted of three exercises, a Body Scan (BS), a hunger exercise (HU), and a satiety (SA) exercise. This study explored how spouses accepted the (dyadic vs. single) training. In a mixed-methods convergence design, the findings of a survey (*n* = 68 couples) and focus groups (*n* = 4) were synthesized. Moderate general acceptance (e.g., regarding feasibility and low burden) and a hierarchical gradient in favor of the BS (e.g., pleasantness and improved sleep quality) emerged. Barriers concerned a perceived lack of the exercises’ usefulness and a limited understanding of the training purpose. A wish for regular feedback and exchange with the study stuff and other participants was expressed. Spousal training involvement was experienced as being rather beneficial. Previously harmonized dietary practices and daily routines appeared as constructive pre-conditions for the joint training. This study highlights the potential and implications of training couples in IS. Future interventions should involve a regular exchange and closer guidance by study staff to promote a better understanding of the processes and goals of IS and IE.

## 1. Introduction

Dietary behavior, such as food choices, is dynamically shaped by a complex interplay of factors, including an individual’s personal state and social as well as (digital) food environments [1,2]. Individual factors comprise biological features, physiological needs (e.g., metabolism, genetic predisposition, and hunger), and psychological components (e.g., personality and emotions), which have been shown to be central for the development of maladaptive eating behavior as restrained or emotional eating [1,3]. On the macro level, socio-cultural factors include normative ideas and discourses (e.g., body image and dieting) and economic as well as political interests (e.g., food marketing and food policy and regulations) [1,4]. In turn, such factors manifest in specific food environments that encompass, for instance, food availability, affordability, and accessibility [4,5]. A prominent food environment, particularly prevalent in high-income countries, is the so-called obesogenic environment, which is characterized by a high density and proximity of (fast) food outlets and easy access to energy-dense, ultra-processed foods [6,7]. Embedded in such external food environments, the individual’s immediate social environment also plays a crucial role in shaping their dietary practices—including (mal-)adaptive eating behavior—throughout the entire life course [8]. In (later) adulthood, the couple relationship is a specifically focal socialization context, in which lifestyle behaviors in general and dietary practices in particular are (pre-consciously) shaped, negotiated, and (re-)established [9].

Both the food and social environment may be in tension with a favorable diet, e.g., by constant (over)consumption stimuli. Against this background, the concept of intuitive eating (IE) has gained scientific interest as a critical response to dieting [10]. IE is an adaptive eating behavior characterized by an attunement to one’s hunger and satiety signals. IE involves trusting the body’s needs, following unconditional permission to eat rather than a restrictive approach to food, and monitoring and reacting to the effects of food on the body (so-called body–food–choice congruence) [10,11]. Hence, the concept of IE emphasizes eating with an intentional focus on internal cues rather than responding to external cues, which may include food availability, sensing food, served portion sizes, or social settings, where eating is encouraged or the norm [12]. Eating in an intuitive manner requires the innate yet often unlearned ability to perceive and process internal bodily signals, which is referred to as interoceptive sensitivity (IS) [13,14]. Previous research suggests a positive association between IS and IE [15,16]. Hence, IS training might be a first step in effectively promoting IE. IS differs considerably among people [17] and was found, for instance, to be lower in individuals with overweight and obesity [18,19] and to decline with increasing age [20].

Previous correlational research suggests substantial associations between IE and various physical and psychological health indicators [21], thereby supporting its adaptive properties [15]. Positive correlations were found, for instance, between IE and life satisfaction, a positive body image, self-esteem, self-efficacy, and health-related quality of life [11]. In addition, IE is negatively associated with maladaptive eating behavior, such as restraint eating, emotional eating, and eating disorder symptomatology, as well as BMI [21,22]. Despite the scarce body of evidence concerning older age groups, one study examined women aged 60–75 years and replicated the positive relationships between IE and psychological health indicators [23].

So far, IE has mainly been researched by observational studies, while only few interventional studies have addressed this topic [24]. Overall, the existing research indicates positive effects of IE on (adaptive) dietary practices and psychological health indicators [25,26,27,28]. However, the state of evidence is sparse and rather inconsistent [25,26,29], suggesting further research directions: a systematic review highlights the need for more research on IE with longer follow-up periods to analyze long-term changes in dietary behavior and diet quality after participation in an IE intervention [25]. Moreover, previous research has mostly focused on younger adults [30,31,32] and the interventive effects of IE in the context of obesity [19], maladaptive eating behavior (e.g., binge eating disorder) [27], or among chronic dieters [33]. Until now, investigating IE in general populations and, more specifically, in middle-aged and older adults and those without diet-related disorders [27,34,35] has been neglected. Along with this, a lack of qualitative explorations of people’s attitudes towards and experiences with practicing IE has been stressed. Currently, there are only a few qualitative studies exploring general populations. Findings from Van Dyk et al. [36] and Erhardt et al. [37] both underline the nature of IE as a dynamic learning process. According to their findings, a central barrier for IE lies in the overcoming of dietary habits and the unlearning of the “conditioning” towards pre-established non-IE practices (e.g., stimulated by the food environment). Both requires discipline in resisting temptation, as well as time and flexible routines. As the authors conclude, there is a further need to explore people’s experiences with (re-)learning IE [36,37]. The study of the present paper aimed to do both, training people to increase their interoceptive sensitivity (IS) as a prerequisite for IE as well as exploring how such training is experienced.

The immediate social context plays an important role in (re-)learning and changing health-related behaviors, including IE. Evidently, individuals in close (e.g., spousal) relationships influence the IE practices of their significant others [37,38]. More generally, spouses’ health-related behaviors have been found to be highly similar, with partner concordance increasing over the course of the relationship [39,40]. This also holds true for dietary practices, i.e., couples tend to synchronize or converge their dietary preferences [41] and food choices [42]. Dietary convergence involves maladaptive eating behavior (e.g., emotional eating [43,44]) as well as favorable [45] dietary styles. However, the extent to which spouses influence each other with regard to IS and IE is still little researched.

Therefore, targeting couples may enhance the effectiveness of health behavior change interventions [37,41]. Previous studies point at the positive effects of couple-based interventions, mostly addressing specific health-related behaviors associated with diet-related illnesses (e.g., diabetes [46]). Yet, the evidence remains largely sparse and inconsistent [46,47,48]. There is, thus, a need for further studies on couple-based interventions targeting overall health-related behaviors independent from ill-health [47]. By doing so, in-depth knowledge can be gained about the mechanisms influencing the success of such interventions. More specifically, an understanding of how couple dynamics and joint everyday practices affect the implementation of interventions could provide important insights. However, the acceptance of couple-based interventions has not yet been sufficiently researched.

In general, there are ambivalent and inconsistent definitions, conceptualizations, and operationalizations of acceptance within the intervention research field [49]. Against this background, Sekhon et al. [49] stressed the lack of systematic theory-based acceptance analyses in the context of healthcare interventions and developed the Theoretical Framework of Acceptability (TFA), which consists of eight components [50]. The present study was guided by the TFA and, accordingly, refers to the authors’ proposed definition of acceptability as “[…] a multi-faceted construct that reflects the extent to which people delivering or receiving a healthcare intervention consider it to be appropriate, based on anticipated or experienced cognitive and emotional responses to the intervention” [49] (p. 4). In this definition, the authors refer to *acceptability*. While acceptability refers to an a priori judgment before exposure to an intervention, the term acceptance describes an a posteriori judgment thereof [51]. In the current study, we analyzed past experiences with the intervention and, hence, unlike Sekhon et al. [50], refer to *acceptance*.

To the authors’ knowledge, this is the first mixed-methods acceptance study on a dyadic interoception-based pilot randomized controlled trial (RCT) to increase IS. The major aim of the current study was to analyze participants’ overall acceptance of the training, as well as its implementation and (non-)continuation into everyday life [52]. Further aims, specific research questions, and hypotheses of the pilot RCT are described in the study protocol [52]. In the context of the present article, we focus on the following exploratory research questions:

- To what extent do partnered adults aged 50 years and older accept an (couple- vs. single-based) experimental interoception-based training program promoting IS?
- What role does the couple context play in the experience, conduction, and post-intervention continuation of the intervention exercises?

## 2. Materials and Methods

### 2.1. Intervention

#### 2.1.1. Intervention Design

The aim of the intervention was to increase IE via training interoceptive sensitivity over 21 days. The training consisted of three interoception-based guided audio exercises. The intervention was a pilot RCT with three measurement points (T0: pre-intervention, training period, T1: post-intervention, and T2: 4-week follow up). We applied a three-arm study design to compare two intervention groups with a control group: *Group 1: Couple-based training* (Index person (G1-I) and partner (G1-P)) vs. *Group 2: Training alone* (Index person (G2-I) and partner (G2-P)) vs. *Group 3: No training* (Index person (G3-I) and partner (G3-P)).

#### 2.1.2. Intervention Description

The training is described in detail in the study protocol [52]. At T0, the participants of G1-I/G1-P and G2-I received a 20-min introductory video about the intervention (study, training, and theoretical background), which they had to watch at home before starting the training. The training consisted of three interoception-based guided audio exercises (Week 1: Body Scan (BS), week 2: hunger (HU) exercise, and week 3: satiety (SA) exercise). The participants had to perform each exercise once a day for 7 days, resulting in a total training period of 21 days. The first exercise was a classic 20-min Body Scan. The exercise was based on a script by Kabat-Zinn and Valentin [53] and was modified according to Fischer and colleagues [54]. The second and third exercises (HU and SA exercises) were based on the IE workbook by Tribole and Resch [55]. In the 8-min HU exercise that was performed before a meal, the participants had to mindfully focus on the perception and quality of hunger signals (e.g., location, intensity, and sensation). In the 9-min SA exercise that was performed after a meal, the participants had to mindfully focus on the perception and quality of satiety signals (location, intensity, and sensation). The aim of the training was to target self-related processes (interoceptive awareness, self-efficacy, self-critical rumination, and self-monitoring), as well as aspects of emotion regulation and attentional control [52,56]. The participants were not instructed to continue to perform the exercises after the 21-day training period.

### 2.2. Acceptance Study Design

For the purpose of the mixed-methods acceptance study, we utilized a convergent synthesis design to integrate the findings from the quantitative data and qualitative focus groups. The research process followed established criteria for mixed-methods studies [57] and was facilitated by the Pillar Integration Process (PIP) (see Section Mixed-Methods Convergent Synthesis) [58]. The reporting complies with the GRAMMS (Good Reporting of a Mixed-Methods Study) guideline [59].

#### 2.2.1. Operationalization of Intervention Acceptance According to the TFA

To facilitate a comprehensive exploration of the participants’ training acceptance, this study and, in particular, the theory-based mixed-methods analysis were oriented towards the TFA [49]. According to this framework, intervention acceptance comprises eight components, which Sekhon et al. [50] operationalized as follows: *Affective Attitudes* address how an individual feels about the intervention, for example, in terms of liking or comfort. *Burden* refers to the amount of effort required to participate in the intervention, including efforts to adhere with the intervention measures. The fit of the intervention with a participant’s moral beliefs or ethical value system is another acceptance component referred to as *Ethicality*. *Intervention Coherence* describes the extent to which a participant understands how the intervention works and/or what it aims for. Another central component is called *Perceived Effectiveness*, i.e., an individual’s perception of the extent to which the intervention has achieved its intended purpose. *Self-Efficacy* refers to the participant’s confidence that they can perform the behavior(s) required to (successfully) participate in the intervention. Another TFA component is called *Opportunity Costs* and refers to the extent to which engaging in the intervention interfered with the participant’s other priorities. More specifically, it addresses the benefits, gains, or values potentially relinquished by participating in the intervention. Finally, the TFA suggests an investigation of *General Acceptance*, i.e., an overarching subjective evaluation of the intervention as a whole.

#### 2.2.2. Recruitment and Organization Procedure

QUAN: This pilot RCT is a sub-study of the larger, ongoing, web-based prospective NutriAct Family Study (NFS) that investigates the epidemiological, psychological, and sociological perspectives on food choices in families. For the NFS, we recruited study participants in groups of two or more family members (spouses and siblings) aged 50+. For further information regarding the study design of the NFS, please see [60]. Eligible for participation in the present study were heterosexual cohabiting couples aged 50+. The aim of the intervention recruitment strategy was to identify people with a low level of IE and invite them to take part in the intervention study with their partner. Therefore, we followed a selective prevention approach. Based on the already available data of the NFS, we successively recruited our intervention sample based on the Intuitive Eating Scale-2 (IES-2) [10] rank, starting with those persons who showed the lowest level of IE. First, we contacted these people (index persons) by mail and invited them to take part in our intervention study together with their partners. If an index person and the partner were willing to participate in our intervention study, we clarified the inclusion and exclusion criteria by phone. From November 2020 to September 2021, we recruited the intervention sample.

QUAL: Three months after the intervention was completed, we recruited the qualitative subsample to build four focus groups. Recruitment took place from May to August 2021. Index persons were contacted via phone to ask for their readiness to participate. In the case of agreement, the participants received an email with detailed study information about the content and practical organization of the group discussions, as well as about measures taken to secure data protection. Participation was compensated with an incentive of EUR 20 per person. Initially, we aimed for group sizes between 5 and 8 people. However, due to the COVID-19-pandemic, the group sizes were limited to a maximum of four people. To facilitate comparative analyses, the focus groups were constituted by (1) women only (G1/2-I), (2) men only (G2-I), (3) couples, where both partners had participated in the intervention (G1-I and G1-P), and (4) men and women who had participated in the intervention without their partners (G2-I).

#### 2.2.3. Data Collection

##### Procedure: Separate, Subsequent Data Collection

QUAN: At each of the three measurement points, we assessed a web-based survey that was filled out by both partners. Furthermore, the pre-assigned index person of each couple was invited to the laboratory and objective variables were assessed. For further evaluation of the acceptance and impact of the training, training evaluation sheets were filled out (paper and pencil) after every week of the training. A detailed flow chart of the intervention design can be found in the study protocol [52]. The trial was registered at the German Clinical Trials Register (DRKS), no. DRKS00024903. The index persons received EUR 10 for each assessment in the laboratory to compensate for travel expenses. For further information regarding the intervention design, please see the study protocol [52].

QUAL: The focus group discussions also took place at the German Institute of Human Nutrition Potsdam-Rehbruecke. Immediately before the discussions, the participants obtained verbal study information, were asked for permission to be audiotaped, and given room for potential open questions. Thereafter, all participants handed in their written informed consent. The recordings were encrypted and stored on a secured institutional drive. The audiotapes were transcribed verbatim and then pseudonymized and anonymized in accordance with the DSGVO (EU 2016/679).

##### Measures

Data were collected separately for the QUAN and QUAL study parts. In the following section, we will only report those measures that are relevant to our research questions. All further measures included in the RCT are described in the study protocol [52].

QUAN: Intuitive Eating Scale-2 (IES-2)

The German IES-2 [11] was applied to measure the level of IE. The IES-2 was included in the web-based surveys. For the current analysis, assessment of the IES-2 at T0 was used. The IES-2 has 23 items and consists of 4 subscales measuring different facets of IE. The items were answered on a 5-point Likert scale ranging from *strongly disagree* (=1) to *strongly agree* (=5). For the current analysis, only the mean values of the IES-2 were used. Higher mean values on the IES-2 reflect higher levels of IE. Previous validation studies have supported the scale’s validity and reliability (e.g., [10,11]). In the current study, Mc Donald’s Omega was 0.80.

QUAN: Training Evaluation Sheets (TES)

The participants of G1-I and G2-I completed three training evaluation sheets (one sheet per exercise) after each week of training. The self-constructed items measured how the participants evaluated different aspects of the exercises. For the current analysis, we used the following items: TES_1) *Please evaluate the exercise regarding the following characteristics*: exertion (TES_1_1), easy to follow (TES_1_2), comprehensibility (TES_1_3), pleasantness (TES_1_4), liking (TES_1_5), usefulness for everyday life (TES_1_6), and easy to concentrate on (TES_1_7). The items were rated on a 5-point semantic differential scale, with higher values representing a better evaluation. To assess the overall evaluation of each exercise, we calculated the mean value of acceptance with the 7 items of TES_1 (Mean_TES_1_BS; Mean_TES_1_HU; and Mean_TES_1_SA). TES_2) *Will you continue to perform the exercise in your everyday life after the end of the study?* The answer options were *yes* or *no*.

QUAN: Acceptance-Related Variables (ARVs)

We further assessed ARVs with self-constructed items. For the current analysis, we used the following items at T1 in G1-I, G1-P, and G2-I: ARV_1) *Overall, do you feel that you can better perceive hunger and satiety signals because of the training?* The answer options ranged from 1 (*not at all*) to 6 (*very much*). ARV_2) *Would you recommend the training?* The answer options ranged from 1 (*not at all*) to 6 (*absolutely*). The following item was assessed at T1 only by G1-I and G1-P: ARV_3) *Imagine if you could do the training again: Would you prefer to do the training again with your partner or alone?* The answer options were 1 (*with my partner*) and 2 (*alone*). The following item was assessed at T1 only by G2-I: ARV_4) *Would you have preferred to do the training together with your partner?* The answer options were 1 (*yes*) or 2 (*no*). The following items were assessed at T2 in G1-I, G1-P, and G2-I: ARV_5) *Have you noticed any changes in your everyday life as a result of the exercises after completing the training?* The answer options were *yes* or *no*. If the participants answered *yes*, they were then asked to specify which changes they had noticed. ARV_6) *Have you continued to perform the exercises after the end of the study?* The answer options ranged from 1 (*no*) to 6 (*once a day*).

QUAL: Focus Group Discussions (FG)

A semi-structured guideline was developed to stimulate discussion and support the exchange of experiences among the participants. The guideline development was informed by the tentative descriptive results of the training evaluation sheets (*QUAN: Training Evaluation Sheets (TES)*). The question stimuli focused on the acceptance of the entire intervention, as well as on specific experiences with the three training exercises over time, i.e., both during the intervention period and after the completion of the intervention. Discussions were also stimulated with respect to the couple context, e.g., the influence and support of the partner in carrying out the exercises. To stimulate a conversation dynamic, the introductory stimulus concerned dietary practices in general, irrespective of IE and the intervention. For this purpose, a quote by a previous study participant was shared: *“Look, that [(his dietary practice)] can’t possibly be healthy. I’ve had three cups of coffee and so many cigarettes before I even eat anything. That’s my weak spot. […] I want to get to the point where I eat at least two pieces of toast and an egg in the morning. I do that occasionally and it even makes me feel better. But I always have to torture myself.”* The participants were then asked to talk about the extent to which they could identify themselves with this statement and in how far potential attempts to change dietary practices had already succeeded/failed—both as individuals and as a couple.

#### 2.2.4. Data Analysis

The data analysis took place in a two-staged process: First, the quantitative and qualitative data materials were analyzed separately. At the end of this parallel analysis process, several meetings took place between the qualitative and quantitative researchers in order to enable a first overview of the respective findings for triangulation, as well as for validation purposes. In the second step, a mixed-methods data analysis was carried out using a convergent synthesis approach [61].

##### Separate, Parallel Data Analysis

QUAN: The quantitative data were analyzed in a descriptive manner. Furthermore, to compare which exercise was evaluated best (Mean_TES_1_BS vs. Mean_TES_1_HU vs. Mean_TES_1_SA) and how the specific evaluation aspects (TES_1_1–TES_1_7) differed between the three exercises, we performed Friedman’s ANOVA with the data of G1-I and G2-I. To compare the recommendation of the training (ARV_2) between the different groups (G1-I, G1-P, and G2-I), we performed a Kruskal–Wallis test.

QUAL: The qualitative data material was analyzed following the Qualitative Content Analysis by Kuckartz [62] using MAXQDA (version 22.0.1). In an iterative process, categories were inductively and deductively derived by the means of “open coding”: in the first step, themes were inductively identified in the transcripts and grouped into so-called “natural categories”. These were then reviewed by a second coder and, where necessary, regrouped until consensus was reached. In the second step, these categories were further differentiated into “analytical categories”, elaborated upon into sub-categories and revised intersubjectively. The material was subsequently fully re-examined and, where applicable, coded within the established category system. Finally, the category system was adapted according to the given research question. This involved deductive coding, a reduction in the system to the most relevant categories, and a partial rearrangement of (sub-)categories. During the entire analysis process, preliminary findings were continuously discussed within the research team, as well as presented and interpreted (multiple times) in a research colloquium to further ensure inter-coder-reliability.

##### Mixed-Methods Convergent Synthesis

The mixed-methods approach was informed by the key features put forward by Creamer [61]. In terms of *Priority* (see [61]), we proceeded in an explorative sequential way, whereby qualitative data were analyzed in greater depth and breadth and the survey results were oriented towards the inductively generated qualitative categories (see PIP, Multimedia Supplementary S1). In doing so, the primary aim of the mixed-methods (MM) analysis was to use the qualitative findings to explore and explain the quantitatively assessed outcomes. The *Timing* of the data collection was separate and sequential for the data collection, as well as parallel during the data analysis. To varying degrees, *Integration* took place at each of the six stages of the research process, all of which Creamer proposes as crucial elements of a Fully Integrated MM Design. *Integration* took primarily place on the analysis and interpretation levels, with the latter aiming at drawing so-called meta-inferences [61].

For the synthesis, we utilized the Pillar Integration Process (PIP), which is an analytical technique for systematically integrating qualitative and quantitative results by the means of a joint display [58]. Joint displays provide visual tools to both integrate and represent mixed-methods results to derive meta-inferences [58,63]. Following the PIP, the synthesis was conducted in four subsequent stages: in Stage I (listing of raw data), we listed selective (only those with relevance to the research question) qualitative categories, codes, and respective interview quotes in the joint display. Subsequently, we matched the (Stage II) quantitative results to the qualitative categories. This step first necessitated the “qualitization” (see [61]) of the quantitative numeric data into the qualitative categories. After the initial matching, a second and a third round of matching were conducted, with attributions based on both the QUAN and QUAL results. Each matching round was guided by the constant comparative method, i.e., the focus was on identifying similarities as well as contrasts. If no match was identified, the respective section was left blank.

Stage III involved the checking of the matched results based on the raw data (quantitative and qualitative data material). In doing so, overlaps were identified, categories were renamed and logically rearranged, and the most relevant raw data (numeric and quotes) were selected and harmonized. In Stage IV, the Pillar Building was conducted, whereby themes were derived equally from the quantitative and qualitative results and combined into meta-interferences. This last analytical step did not only involve inductive theme development, but also the deductive derivation of inferences drawing on the TFA. The entire integration process was iteratively conducted by two researchers experienced with qualitative evidence syntheses.

## 3. Results

### 3.1. Sample Characteristics

QUAN: We enrolled *n* = 71 heterosexual couples into the study. One couple and one partner dropped out before the groups were allocated and two couples and one partner dropped out before the training started, resulting in *n* = 68 couples and, in total, *N* = 134 participants. The total sample consisted of *n* = 68 women (50.7%). The mean age was *M* = 67.4 years (*SD* = 5.6, range = 53–85 years). Table 1 displays the sample characteristics of the QUAN data separately for each group.

QUAL: The final sample for the focus groups consisted of twelve individuals. Among the participants, 50% were women. The mean age of the participants was *M* = 65.9 years (*SD* = 4.9, range = 60–78 years) and the mean BMI was *M* = 24.1 kg/m^2^ (*SD* = 2.5, range = 20.3–28.3 kg/m^2^).

### 3.2. Main Results

As a major result of the MM convergent synthesis, a pillar consisting of six overarching themes was developed, which combined the quantitative (“qualitized”) and qualitative categories. The QUAN categories primarily concerned aspects of practicability, ease of realization, and the integration into everyday life. Among other things, the QUAL categories provided insights into the participants’ lived experiences, attitudes towards the training and the concepts of IE and mindfulness in general, and explained reasons for a perceived (lack of) training effect. The pillar themes reflected six out of the eight TFA components proposed by Sekhon et al. [50], which were empirically derived throughout the synthesis process. Each of these components appeared several times, e.g., for the training exercises. The two TFA components of *Ethicality* and *Opportunity Costs* did not occur in our data. The most significant material, which primarily emerged from the FG, concerned the TFA components of *Perceived Effectiveness* and *Affective Attitude*, indicating their particular importance for the training acceptance.

**Figure 1:**
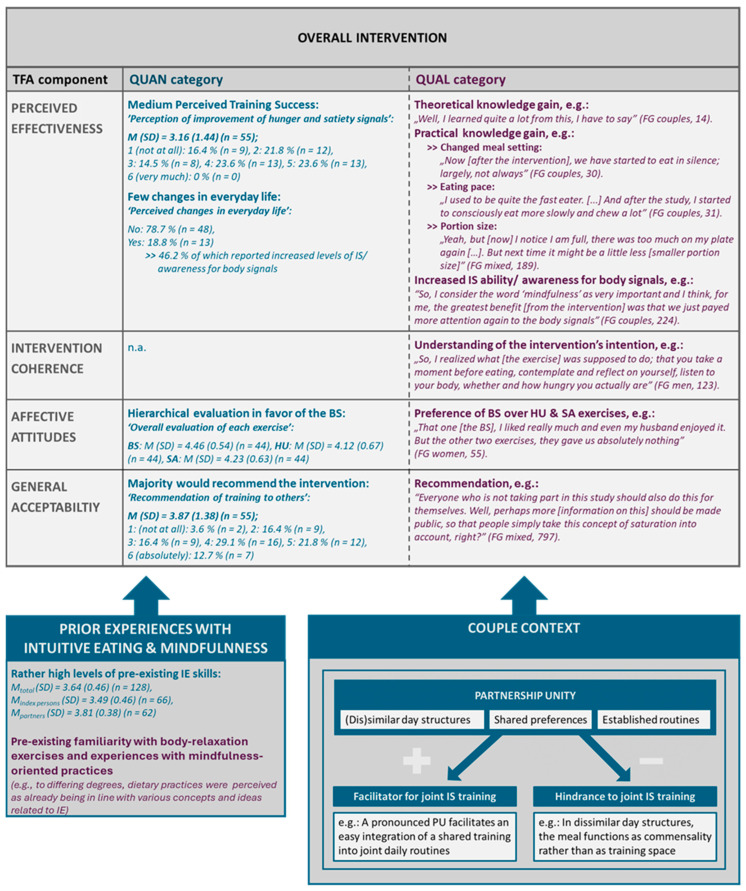
Overview of selected results regarding the overall intervention acceptance and main context factors. Note. QUAN = quantitative, QUAL = qualitative, FG = focus group, M = mean, SD = standard deviation, IE = Intuitive Eating, IS = Interoceptive Sensitivity, BS = Body Scan, HU = hunger exercise, SA = satiety exercise, and PU = Partnership Unity. This figure contains selected results on the overall intervention acceptance. For more detailed information on the acceptance of the respective exercises and respective TFA components, as well as further primary data (QUAN/QUAL), please see Multimedia Supplementary S1 (PIP).

In Multimedia Supplementary S1, a joint display is depicted showing the results of the Pillar Integration Process (PIP). The joint display provides a detailed overview of the pillar themes, and the inductively generated categories are depicted for the overall intervention, each training exercise, and the couple context. Figure 1 illustrates a condensed overview of the selected results regarding the overall intervention acceptance and the main context factors shaping the participants’ acceptance.

Subsequently, the results are described for each of the identified TFA components in Section 3.2 (see Section 3.2.1, Section 3.2.2, Section 3.2.3, Section 3.2.4 and Section 3.2.5). In addition, Section 3.3 deals with findings regarding the couple context, which could be reconstructed inductively on the basis of the QUAL FG beyond the TFA-based acceptance analysis. Section 3.3.1 deals with the extent to which the partner and/or a joint participation was perceived as a benefit in carrying out the exercises and achieving the intended training effects. Section 3.3.2 addresses the dynamics of the couples’ everyday lives that were identified from the FG, allowing conclusions to be drawn about the barriers and facilitators for joint training execution.

#### 3.2.1. Affective Attitude

Our findings document a hierarchical gradient regarding affective attitudes towards the three exercises: the Body Scan was preferred over the hunger and satiety Exercises, which is reflected in both the qualitative and the quantitative results. The quantitative findings showed relatively high values for pleasantness (BS: *M* (*SD*) = 4.56 (0.79), HU: *M* (*SD*) = 4.05 (0.91), SA: *M* (*SD*) = 4.32 (0.83)) and moderately high values for liking (BS: *M* (*SD*) = 4.23 (1.08), HU: *M* (*SD*) = 3.61 (1.17), SA: *M* (*SD*) = 3.75 (1.28)) for all three exercises. Insights gained from the focus groups allowed us to further understand these outcomes: the participants expressed a relatively pronounced general enjoyability and pleasantness associated with the BS. This also applied to cases where initial skepticism was present, but which gave way to a positive attitude during the course of the training, as one participant described with respect to her husband:

*“But [then] he was really pleased by the Body Scan. […] At the beginning, he said ‘that’s hocus-pocus’ or something like that and I said ‘why don’t you try it first’, yeah? And then [(afterwards)] he said: ‘that was really pleasant!’ […] [So,] I really much liked [the BS] and even my husband enjoyed it. But the other two exercises, they gave us absolutely nothing.”*

(FG women, pos. 55/68)

Despite this hierarchical evaluation in favor of the BS, the participants also showed gratitude for the knowledge gain and heightened meal anticipation elicited by the HU exercise:

*“At the end [of the HU exercise] I always told myself: ‘So, now you can have a delicious dinner!‘. […] And then I was really looking forward to eat something and actively went upstairs saying: ‘Oh, now you are allowed to eat something really nice!’.”*

(FG mixed, female participant, pos. 445)

Concerning negative attitudes towards the exercises, the qualitative and quantitative findings were somewhat divergent. For instance, while (rather) high levels of pleasantness were reported for the HU, negative affects played a greater role in the group discussions: Here, the participants discussed unpleasant feelings such as annoyance, disappointment, and anger, especially with the hunger and satiety exercises, but also regarding the Body Scan—albeit to a lesser degree. These feelings were often placed in the context of a perceived “needlessness” or “senselessness”, as illustrated by the following quote:

*“But this hunger exercise and also the satiety exercise, those drove me up the wall, because the questions asked were complete nonsense in my point of view. Like, where do I feel hunger‘. […] I feel it in my stomach and […] in the digestive tract. And satiety, too. But I don’t feel it in my hands and feet—so, these were completely illogical questions [laughs]. Practically every time these questions came—It was over. That’s when I got annoyed.”*

(FG men, pos. 92)

#### 3.2.2. Burden and Self-Efficacy

To varying degrees, *Burdens* were identified with respect to each of the three training exercises, whereby this TFA component appeared to largely overlap with aspects related to *Self-Efficacy*. Therefore, both themes were conjointly addressed in the PIP and subsequently. According to both the quantitative and qualitative findings, the BS required low levels of effort (BS: *M* (*SD*) = 4.71 (0.70)) and was evaluated as easy to follow (BS: *M* (*SD*) = 4.67 (0.64). The associated relatively high degree of perceived self-efficacy may partially be explained by the participants’ prior experiences with other kinds of relaxation exercises, such as the regular Qigong practice mentioned by one participant. Regarding the HU and SA exercises, low levels of effort (HU: *M* (*SD*) = 4.51 (0.82), SA: *M* (*SD*) = 4.70 (0.59)) were reported and the exercises were evaluated as easy to follow (HU: *M* (*SD*) = 4.67 (0.56), SA: *M* (*SD*) = 4.79 (0.47)), while some burdens and little experiences of self-efficacy were revealed in the group discussions. For example, the participants perceived the exercises as challenging, as they were not used to sensing their hunger and satiety signals: *“[…] It was exhausting because I didn’t realize if I was full or not, what is that feeling?”* (FG mixed, male participant, pos. 431). The satiety exercise appeared to be more difficult to perform, partly because the feeling of satiety was reported to be more subtle and, therefore, harder (if at all) to sense as compared to hunger: *„[…] and maybe I had more of a problem with the satiety exercise. So, hunger is easier to identify for me than satiety”* (FG couples, male participant, pos. 288). In spite of these difficulties, however, repeated practice of the exercises seemed to have a positive influence on the level of self-efficacy over time. The participants expressed their experience of a familiarization effect, making the exercises easier as the training progressed, as this quote illustrates: *“Well, you first had to get used to finding a way in, because you—it [the HU exercise] was unfamiliar. But it got better over time […]”* (FG mixed, pos. 430).

#### 3.2.3. Perceived Effectiveness

In respect to the *Perceived Effectiveness* concerning the overall intervention, the quantitative and qualitative results were inconsistent again. While the quantitative data displayed a relatively low perceived training success (*M*_ARV_1_ (*SD*) = 3.16 (1.44)), with more than three-quarters of the participants (78.7% (*n* = 48)) reporting no associated changes in everyday life, the qualitative results indicated rather positively perceived training effects. In particular, the qualitative analysis revealed two different (but related) types of knowledge gain: on the one hand, the participants shared their perceptions in terms of a theoretical knowledge gain, i.e., of having learnt something new about the concept of mindfulness and/or IE. On the other hand, and seemingly of greater relevance, different types of practical knowledge gains towards more conscious eating practices were discussed in the FGs. These mainly concerned a general greater awareness towards (diet-related) internal body signals. In some cases, this was rather framed as a re-learning or intensifying of already existing skills, rather than establishing new ones:

*“So, I consider the word ‘mindfulness’ as very important and I think, for me, the greatest benefit [from the intervention] was that we just paid more attention again to the body signals.”*

(FG couples, female participant, pos. 224)

Moreover, various changes in mealtime routines and eating habits were mentioned, such as a quieter meal setting, smaller portion sizes, and a slower eating pace, as illustrated by the following quote: *“I used to be quite the fast eater. […] And after the study, I started to consciously eat more slowly and chew a lot”* (FG couples, male participant, pos. 31). Interestingly, although these changes (e.g., increasing the amount of chewing) were not explicitly addressed as part of the training exercises, they were apparently associated with IE and considered to be integral to the individual intervention success.

In comparison to the other exercises and particularly to the SA exercise, the Body Scan was perceived as the most useful for everyday life. As the FG revealed, its useful benefits included a better ability for interoception, stress reduction, and a general calming and relaxation effect—both physically and mentally—as well as an improvement in sleep quality. One participant recalled his positively experienced effects of the BS exercise as follows:

*“It was the first time I consciously felt my body in every corner, right? And then I lay down for another moment and afterwards I got up, let‘s say, as light as a feather; I was calm, also calm inside, and found it really fascinating what it [the BS exercise] does to your mind.”*

(FG Mixed, male participant, pos. 157)

At the same time, a few participants also criticized a lack of effect and/or were not convinced this exercise could have any kind of influence on their eating practices. While the BS showed a fairly high perceived usefulness for everyday life overall (*M* (*SD*) = 3.87 (1.12)), both the HU and SA exercises were perceived as only moderately effective (HU: *M* (*SD*) = 3.29 (1.12), SA: *M* (*SD*) = 3.09 (1.14)). On the one hand, a large number of participants reported no (lasting) training effects. This may be explained by findings of the participants’ pre-existing mindfulness-oriented lifestyle in general, and mindful or IE-related dietary habits in particular. Both of these, as expressed in the FGs, seemed to prevent participants from experiencing training effects:

*“I would actually like to emphasize [that] the- the [HU] exercise itself didn’t give me anything, because I already live according to this pattern anyway, right?”*

(FG men, pos. 187)

*“So, for us [couple], the meal is a ceremony in a certain sense, right? Not that we make a big fuss about it, but it all happens in a calm manner. We chew thoroughly without counting the amount of chewing actions per bite. But yeah, it all happens in peace.”*

(FG men, pos. 124)

On the other hand, the HU and SA exercises were perceived as being useful due to a gain in knowledge, e.g., the exercises provided new aspects that participants were “*grateful for*” (FG couples, female participant, pos. 89). More strikingly, the participants shared experiences of improved interoception resulting from regular practice of the SA and/or HU exercises. This training effect was not only related to the intervention period, but also to the time afterwards, as this participant remembered:

*“[…] in retrospect, in the time since [the intervention], I have to say I’ve become more aware of it. I realize better when I’m hungry and when I’m full. I’ve become more aware of this feeling. I used to notice it less.”*

(FG mixed, female participant, pos. 178)

With regard to these experiences, however, we found a discrepancy between the objective of IE to improve the sensational attunement to HU/SA signals and the practical realization by the means of “rationality”. In this context, one male participant described a heightened reflexive awareness for the concept of satiety, albeit with remaining difficulties with actually sensing this body signal:

*“What the study did for me: It raised awareness for the concept of satiety. Because I- even though I don’t notice if I am already full at that moment, I might notice it minutes later, but by then, I’ve already had several other forks or spoons. It [now] has rather become a matter of rationality. The concept of satiety has triggered a bit more reason in me, for now I say: ‘No, that’s enough. You might not be really full yet, but that’s enough, stop it.’ Right? That’s what my consciousness tells me.”*

(FG mixed, male participant, pos. 797)

Here, the sensation of satiety was perceived as an automatism and an eating practice closely aligned to the principles of IE, which does not (yet) seem possible (merely) by the means of interoception. Instead, the concept of mindfulness is brought into consciousness on a reflexive level, and the habitual eating practice (of overeating) is disrupted through rational action, as in this case through an auto suggestive imperative.

#### 3.2.4. Intervention Coherence

The extent to which the participants understood how the intervention was supposed to improve their interoceptive sensitivity and/or IE skills was not quantitively assessed. Yet, a few conclusions can be drawn from the qualitative study findings. Albeit in rare cases, the participants seemed to have understood the intended training purpose, as the following quote demonstrates:

*“So, in principle, I realized what it [the HU exercise] was supposed to do; that you take a moment before eating and reflect on yourself, listen to your body and feel how hungry you are.”*

(FG men, pos. 123)

At the same time, ambiguities also became evident, as several questions were raised in the FGs about the intended intervention purpose, for example, why IS was of interest as a research topic. In addition, there appeared uncertainties regarding the concept of interoceptive sensitivity, its specific aims (e.g., as distinct from IE), and its practical implementation by the exercises. Implicitly, this became evident when the participants described IE practices in general, as well as when they put their training experiences in the context of broader mindfulness-oriented lifestyle behaviors and attitudes. While this illustrates a difficulty in separating the overlapping concepts and underlying principles of IS and IE, it may, nevertheless, be an indication of a general understanding of the intervention’s associated aims. More explicitly, however, a lack of *Intervention Coherence* was shown when the participants expressed their desire for feedback, both individually on their own performance (e.g., *“[…] some kind of personalized information”* (FG women, pos. 175)), as well as in comparison to the other participants. The following quote shows exemplarily that this desire was associated with the participants’ ambition for a correct training execution and an accompanying uncertainty: *“I would be interested in whether all the participants are basically marching in the same direction or if we are generally off the mark”* (FG men, pos. 219). This desire for objectifiable, quantified comparison and external validation implicitly challenges the participants’ acceptance in terms of the *Intervention Coherence*.

#### 3.2.5. General Acceptance

General acceptance of the intervention was not directly measured, but could be inferred from several indicators. Foremost, recommendation was used as a proxy, showing that the majority of the participants (*n* = 35, 63.6%) would have (rather) recommended the training to others. However, a considerable proportion also indicated that they would not at all (*n* = 2, 3.6%) or rather not (*n* = 18, 32.8%) have done so. Noteworthy, the recommendation rates were significantly higher among the participants who had completed the training without their partner (G2-I). The qualitative study findings primarily supported a positive view: in the mixed FGs, one participant suggested, *“[…] everyone who is not taking part in this study should also do this for themselves”* (FG mixed, male participant, pos. 787), while another participant emphasized a particular benefit for young people with overweight:

*“[…] Young people should participate in this kind of dietary study, right? Because I know that currently many, well, let me say overweight people, are really, really young. So that might also be an approach: to invite the younger folks to this kind of intervention to make them realize how important this is, especially for later life. Because if they are already that overdimensioned now, how will this be in old age? That is really important, right?”*

(FG mixed, female participant, pos. 36)

This suggestion demonstrates a goal-oriented comprehension of the concept of IE, namely weight reduction. Against this understanding, the participant implicitly positioned herself in contrast to young people with overweight, who she considered to be in particular need of an IE practice, amongst others with respect to healthy ageing. A personal need for IE as an end in itself is, therefore, not considered here. In line with the other participants’ statements, this indicates a certain degree of positive self-assessment regarding a healthy, mindful dietary practice. This is reflected in the study sample’s IE levels (*M*_index persons_ (*SD*) = 3.49 (0.46); *M*_partners_ (*SD*) = 3.81 (0.38)), which are comparable to adults aged 50+ in a German community sample [11]. Moreover, not only was the training itself deemed recommendable, but so was the promotion of the principles associated with IE: *“Well, perhaps more [information on this] should be made public, so that people simply take this concept of saturation into account, right?”* (FG mixed, male participant, pos. 797).

The *General Acceptance* of the training was also reflected in the (partial) continuation of the exercises during the period following the intervention. In the questionnaires, the participants were asked for their intention to continue the training exercises (at T1) and their actual training exercises continuation (at T2). While the HU and SA exercises were not intended to be continued after the intervention by the vast majority (HU: intention yes = 29.5% (*n* = 13), no = 70.5% (*n* = 31); SA: intention yes = 36.4% (*n* = 16), no = 63.6% (*n* = 28)), the participants seemed to have more positive expectations of the BS. Here, 77.8% (*n* = 35) indicated they would possibly continue this exercise. However, 22.2% (*n* = 10) could not imagine themselves doing so.

With respect to the actual training exercises continuation, the quantitative data revealed that approximately half of the participants (54.1%, *n* = 33) did not continue the training at all, while others indicated they had continued the exercises sporadically (18%, *n* = 11), regularly (19.7%, *n* = 12), or in a daily manner (8.2%, *n* = *5*). To further explore which exercises were continued, to what extent, and for what reasons, the qualitative results provide further information:

Congruent with the findings showing a hierarchical exercise evaluation in favor of the BS (also see *Affective Attitudes*, Section 3.2.1), this exercise was continued the most frequently and consistently. At the same time, however, sporadic training exertion was also shared in the FGs: *“Well, I neither do it [the BS exercise] regularly. I somewhat do it as needed”* (FG couples, male participant, pos. 201). Such occasional practice was particularly evident in connection with a purposeful intention set by the participants themselves, such as for improving sleep quality (“[…] *It calms you down, so it’s sleep-inducing, if you can’t sleep*” (FG couples, female participant, pos. 202)). Thus, the experience of specific needs that were believed to be met by practicing the BS seemed to encourage post-intervention continuation of this exercise. In the case of continuation, the participants reported they did so without listening to the audio instructions.

In the case of the HU exercise, there was some continuation from time to time by a rather small proportion of the participants. Similar to the BS exercise, the HU was then performed without listening to the audio file and only particular parts were selected in response to the individuals’ needs and wishes. Moreover, although participants in two FGs reported that aspects of the SA exercise remained “in the back of their minds” (FG couples, female participant, pos. 89), none of the interviewees reported the retention of the SA exercise after the end of the study.

Across all exercises, the reasons for discontinuation included a perceived lack of added value and purpose, which may possibly be related to the level of understanding of the intervention’s intended effects (also see *Intervention Coherence*, Section 3.2.4). This was particularly evident for the HU exercise, as the following statement underlines: *“To this day, I still don’t really see the (deep) meaning of it [the HU exercise]”* (FG men, pos. 91).

Another reason for discontinuation was that the participants saw no need or little value for themselves due to their already pre-existing mindfulness-related skills: *„I wouldn’t say that it didn’t give me anything at all, but I was just already eating more consciously before [the training]”* (FG couples, female participant, pos. 284)) (also see *Perceived Effectiveness*, Section 3.2.3). Conversely, there also appeared to be a perceived inability to perform the exercises—especially regarding the HU exercise. Some participants noted that, particularly with increasing age, they either rarely felt hunger (anymore) or did not experience it at all, which rendered the hunger exercise rather pointless to them: *“Well, […] just like you [(other participants)] don’t know the feeling of satiety, I don’t really know the feeling of hunger either”* (FG women, pos. 128).

Moreover, the aim of the SA exercise was shown to be in conflict with their own habitual eating practices for some. This was especially the case in connection with certain socialization experiences, whereby socio-culturally shaped normative eating rules played a role: *“I was actually raised to always eat up, you know? [laughs]. That‘s why I always finish my plate*“ (FG mixed, male participant, pos. 204). Here, the norm of eating everything on one’s plate seemed to interfere with the principles of IE.

### 3.3. Couple Context

#### 3.3.1. Intervention Benefit of Partner Participation

Regarding the intervention, the qualitative FGs showed a rather beneficial effect of having the partner involved in the training execution. Only occasionally was the partner perceived as a slight hindrance. On the one hand, this was related to incongruent levels of motivation within the couple, which, in one case, for example, required persuasion on the part of the female participant. On the other hand, a greater distractibility and permission to “cheat” was observed as a barrier when performing the exercises together: *“[laughs] if one cheats, the other one cheats too”* (FG couples, female participant, pos. 100). A much more positive emphasis was placed on the joint participation, whereby motivation also played a decisive role:

*“Doing it together always makes it easier.//[male partner:] Exactly. So no, we haven’t slowed each other down, you rather motivate each other.”*

(FG couples, female//male participant, pos. 144)

*“I didn’t need to be motivated by my wife. But it was pleasant to do these two hunger and satiety exercises together with her.”*

(FG mixed, male participant, pos. 417)

As documented in the last quote, there also were participants who explicitly did not need any external motivation from their partner. This may cautiously be explained against the background of the general attitude towards participation in the study: overall, the FGs demonstrated the participants’ aspirations of being conscientious and appropriate in the study involvement. For example, one male participant referred to his *“[laughs] Prussian obedience*” (FG male, pos. 89). More importantly, this shows that partner participation was positively associated with an affective component—particularly with feelings of pleasantness and joy. To a certain extent, joint training participation even appeared to have a protective or facilitative effect. As illustrated by the following quote, for instance, the exercise itself was associated with a “struggle”, which could be counteracted by the commensal practice and exchange of experience: *“When you do it together it’s definitely more fun, obviously, than struggling with it by yourself.//[female partner]: Then you can exchange experiences with one another: ‘How was it like for you?*” (FG couples, male//female participant, pos. 145).

In addition, the partner’s influence was also perceived positively when they were not in the training group (G2-P) and did, therefore, not perform the exercises. Here, the partner was perceived as supportive, e.g., by active encouragement of regular practice. Although the partners of training group 2 were not instructed to support their spouses, this encouragement was driven by the positive training effects observed by the partner: “*Even though he didn’t participate […], it was important to him to remind me because he noticed it was good for me*.” (FG women, pos. 114).

There was no clear quantitative evidence of a perceived benefit from participation as a couple, which we could only analyze based on a proxy measure: when asked about their preferences for future training participation, the vast majority of participants would not change the way in which they participated. Hence, both those who attended with their partner (G1-I/P: preference with partner: 67.6% (*n* = 25), without partner: 32.4% (*n* = 12)) and those who participated alone would like to do so again in the future (G2-I: preference with partner: 38.9% (*n* = 7), without partner: 61.1% (*n* = 11)).

#### 3.3.2. Training Intuitive Eating in Everyday Couple Life

The FGs revealed that the couples’ everyday lives played a decisive role in the conduction and experience of the training exercises. Some relationship-specific dynamics and routines emerged in this context, allowing conclusions to be drawn about couple-related barriers and—to a greater extent—facilitators. Overall, both couples and individuals in the respective FGs demonstrated the existence of an established *partnership unity* (PU) in terms of their general lifestyle behaviors and shared daily routines. This specifically concerned various diet-related aspects such as meal structures and habits, as well as food preferences and choices. The participants attached a normative, positive meaning to their PU (e.g., by referring to themselves being “*well synchronized*” or “*well attuned*”), indicating an ideal picture of a couple relationship (not only) in the context of food. This became particularly evident in distinction to others, as expressed in this example:

*“And so, we have—we are both such a, such a unit. And when it comes to cooking and eating habits, we’re relatively well-adjusted. […] Fortunately, I have to say, I know other examples [laughs]…, we are a pretty good unit when it comes to diet and exercise. So that works out quite well.”*

(FG women, pos. 70)

The perception of a ‘good’ PU was regarded as an important basis or at least a beneficial starting point for practicing the training exercises as a couple. To differing degrees, this positively perceived PU appeared as a result of (ongoing) negotiation and convergence processes. The following quote illustrates the assumed link between a well-established (diet-related) PU and perceived joint training success:

*“So, it’s not a problem for us, it was already the case that we were largely in alignment when it came to food and nutrition, right? […] Well, over time, that has also harmonized. […] In this respect, it wasn’t a problem for us to keep it [the intervention] up together.”*

(FG couple, male participant, pos. 157)

Here, shared dietary practices had been established through mutual negotiations over the course of the relationship. Implicitly, this harmonization process was viewed as a success (or at least the absence of conflict), which the participants relied on as a resource for an unproblematic joint realization of the intervention training program.

As a facilitator or barrier for a positively experienced and successful joint training execution, PU unfolded on several levels, i.e., various aspects of a shared daily life constituted the experience of PU. First, various harmonized diet-related practices such food choices and meal preparation (e.g., also see quote in line 753) appeared in the context of “well-synchronized” PU, which, in turn, was considered to be a constructive pre-condition for joint training success. More specifically, the participants described how their initially (more or less) divergent dietary styles had been subject to a convergence process during the course of their relationship. This concerned ethically and morally motivated dietary styles (e.g., vegetarianism), as well as health-oriented ones (e.g., reduced sugar intake and restricted alcohol consumption). Closely related, some couples also described a gradual alignment in their taste preferences. As an example, in one case, the husband had converged his diet to suit his wife’s medical condition. Proceeding from this first step of adaptation, the wife further perceived the development of a harmonized ‘PU taste’:

*“[…] Nutrition has always played a bit of a role for me because of my illness [(Diabetes)] and (. ) my husband has always supported me in this, in that I’ve put certain things (2)- less on the table [e.g.,] sweetness has been gradually reduced to the point where we are now and we both like it and I don’t have to have a guilty conscience if I bake a cake that isn’t as sweet [laughs] as others (.) would perhaps like it to be. […] [Overall,] we have supported each other well in this respect [(in adjusting dietary preferences)] (.).”*

(FG couples, female, pos. 158)

Besides the aforementioned diet-related practices, (in)congruent daily rhythms were discussed as being formative for PU. Primarily, time and setting issues were emphasized, e.g., in relation to working hours and conditions. Spouses’ similar everyday structures and shared mealtimes were perceived to have a facilitating effect on the joint training realization. For example, working from home simplified the scheduling of a joint exercise session:

*“And of course, we have good conditions [(for a proper training realization)]. […] We are currently working from home, at least I am, due to COVID, and we can really plan our daily routine together now.”*

(FG couples, male participant, pos. 30)

Mostly, however, couples rarely shared their meals during working weeks, with breakfast being an exception. The training (especially the HU and SA exercises) was then sometimes perceived as being an additional burden interrupting their irregular yet coordinated daily routines. This was also the case among couples in which one spouse only participated in the training. Especially when there was less importance and meaning attached to food as compared to leisure activities, for example, commensal meals were less of a priority and only taken if the schedule allowed so. This consequently impeded regular training realization, as shown in the following:

*“I’d say that we actually make the biggest compromises when it comes to hunger and [as far as hunger is concerned], we had paid the least attention to the study. At the weekends, where we always eat together, there are certain times for us—//male participant: yes//where we have something planned, [such as] doing sports sometimes or I go to church or something. So, [at the weekend] our daily routines are pretty similar. And then it’s a compromise as to when the meal fits in. We don’t pay that much attention [to hunger].”*

(FG couples, female participant, pos. 233)

In another way, dissimilar daily “rhythms” appeared as a hindrance, specifically when the meal was one of the few moments of the day that served the purpose of socializing. Here, communicative commensality was prioritized over the training interoception of hunger or satiety signals and other principles related to IE:

*“Well, so far, we haven’t managed to concentrate on eating so much, because we don’t see each other that much. I work, he doesn’t. Erm, and we have more or less the opposite daily rhythm. [laughs] Eh, and for us, mealtimes are more about communication and sharing.”*

(FG couples, female participant, pos. 33)

In contrast, rather than a place of communication, the meal became a place of silence and interoceptive concentration in other couples. Here, achieving a particularly adequate or successful training realization was sought by creating specific facilitation conditions, i.e., the introduction of the rule to eat in silence and/or without speaking:

*“For me, it was like that, [I have learnt]—to eat more consciously or concentrating on eating. […] This calming down, this relaxing and paying attention to what you eat and how you eat—because we also liked to have the radio on for breakfast and then listen to the news or music, […]. And we’ve now abandoned that after the study (.), so to speak. So, we really had breakfast in peace and quiet then.”*

(FG couples, male participant, pos. 21)

*“[…] Well, we have introduced this now [(since the intervention)] as far as possible, not always, that we eat in silence. […] We have realized that it’s good to eat in silence.”*

(FG couples, male participant, pos. 30)

As illustrated by the two above-cited quotes, the training stimulated the impetus to try new and mindful eating practices. Hence, the perceived effectiveness of the intervention training went beyond the intervention’s objectives and exercise instructions. While other couples did not establish such new routines or favorable training conditions, these couples identified rather unfavorable settings that hindered them from practicing the exercises. In particular, the restaurant setting and specific temptations due to a larger variety of foods and bigger portion sizes were experienced as impeding the ability to pay attention to one’s satiety signals.

Moreover, mindfulness-oriented daily routines some couples had already established prior to the intervention were seen as being a particularly favorable precondition. Specifically, a routinized shared meditation practice facilitated the realization of the training sessions and the understanding of the underlying intervention objectives. The following spouses remembered collectively how they individually had made sense of the more general mindfulness-oriented BS exercise as some kind of preparatory part of the intervention:

*“I found it interesting that this Body Scan was part of it [the diet-focused intervention] […]. So, that was nothing new to me. Um and, yes, I have already had my thoughts: Yes, as an introduction to a mindfulness exercise, the Body Scan is of course very good, so that you first get to know this kind of approach.//[female partner]: Yes, I also thought that you should first familiarize yourself with your own body and pay attention to body signals. And then I thought to myself: Yes, of course. When it comes to eating, perhaps that also plays a decisive role (laughs).”*

(FG couples, male//female participant, pos. 222/223)

Interestingly, this couple further shared that their (mealtime) routines had been subject to a past negotiation process, partly because the wife’s diet accorded with principles of intermittent fasting. Among other things, this specific dietary style required finding a compromise regarding the timing of shared meals:

*“Well, we have adjusted to each other a bit over the years, and it usually works out well with the mealtimes. […] We have simply adapted our daily routines over time so that it’s okay for both of us. […] Of course, new aspects were brought in here [(by the training)], but we had already managed it quite well before, I’d say.”*

(FG couples, male participant, pos. 229)

This statement further reflects how the participant viewed the benefits of the training for his and his wife’s PU: On the one hand, the training encouraged the integration of “new aspects” into the couple’s daily life. On the other hand, however, a (more substantial) change was not considered to be necessary due to a previously negotiated, established PU. Notably, the intended training effect of improving interoceptive sensitivity was not addressed here. Instead, however, the training seemed to be understood more generally and associated with an improvement in their everyday couple life.

## 4. Discussion

To the authors’ knowledge, this is the first mixed-methods acceptance study on a dyadic interoception-based pilot randomized-controlled trial (RCT). The RCT aimed to train interoceptive sensitivity (IS) among couples aged 50+ as a crucial first step towards re-learning intuitive eating (IE). The overarching objective of this study was to analyze the participants’ acceptance of the training in general, as well as its practical implementation in everyday life. Two main exploratory research questions were addressed: first, the extent to which partnered adults aged 50+ accepted the (couple- vs. single-based) interoception-based training; and second, the role the couple context played in the experience, conduction, and post-intervention continuation of the training.

While popular as well as scientific research on IE is growing, research on IE and its associated principles—including IS—is still limited to aims that can be achieved by IE (e.g., adaptive eating behavior [22,27,28]) or specific target groups (e.g., individuals with obesity [18,19] or women [30,37,38]). Basic research on the mechanisms involved in practicing IE in general populations is scarce. Against this background, previous work has specifically emphasized the need for qualitative explorations on re-learning IE [37]. Moreover, there is little research on practicing IE that takes the couple context into account, which may, however, decisively shape the ways in which individuals engage in practices of IE [38]. Therefore, our study aimed to investigate a couple- (vs. single-) based intervention. Addressing these research gaps, the present acceptance study provides first insights into the role the couple context plays, e.g., in promoting or inhibiting joint IE training execution.

### 4.1. Training Acceptance

Overall, our synthesis revealed a fairly moderate *General Acceptance*, whereby the training, in general, was perceived as feasible and associated with a relatively low burden. However, a mixed picture emerged, particularly with respect to the *Perceived Effectiveness* and *Affective Attitudes*. Here, a hierarchization of the more general mindfulness-based Body Scan (BS) over the hunger (HU) and satiety (SA) exercises became evident. Besides a few exceptions, the BS was primarily evaluated as pleasant, enjoyable, and useful for everyday life. The BS was associated with various beneficial effects even apart from eating, such as an improved sleeping quality. Some of these positive experiences associated with the BS in particular were also shown in an earlier study about women who had been practicing IE over a minimum of 1.5 years without an intervention context [37]. For instance, a greater “headspace” and an improved awareness of body signals was described.

As in comparison to the BS, the HU and SA rather evoked negative emotions and were associated with a medium *Perceived Effectiveness*. In addition, the participants expressed some disappointment with the HU and SA training content and reported a lack of training success on various levels. This ranged from mostly slight difficulties with the exercise implementation (both organizationally (e.g., due to the spouses’ incongruent daily routines) and in terms of content (e.g., perceived lack of interoceptive ability)) to, in extreme cases, anger about the training content. In these cases, the training experiences, thus, rather fell short of the intended intervention objective.

The hierarchical exercise evaluation may have been related to the participants’ pre-existing familiarity with body relaxation exercises and their experiences with mindfulness-oriented practices and lifestyles in general. In contrast, the HU and SA exercises were novel and (mostly) surprising to the participants. Closely related, our findings suggest a partially insufficient understanding of the objectives and intended effects of the HU and SA exercises, resulting in a more negative evaluation compared to the BS. This can be further explained by the quite high popularity of mindfulness-based stress reduction programs that include the Body Scan. The implications of these results are described in Section 4.6.

Compared to German adults aged 50+ [11], our participants showed similar pre-existing levels of IE, which is related to the sampling strategy (see Section 2.2.2). In addition, they demonstrated relatively pronounced mindfulness and health-oriented lifestyles. As an example, they saw their dietary practices as already being in line with various IE ideas to differing degrees, rendering the HU and SA (slightly) obsolete.

Concerning the TFA dimension of *Intervention Coherence*—i.e., the participants’ understanding of how the intervention aimed to increase IS—a thin line of conflict between a more reflective awareness and intuitive practices could be observed: for example, for one participant, the training did not lead to an increase in IS, but rather to a stronger pronunciation of rationality regarding the concept of satiety. This style of eating is described as “flexible control” [65]. On the one hand, raising awareness for the concepts of hunger and satiety could be seen as a necessary step or preliminary stage in increasing IS. On the other hand, however, eating guided by control, instead of by one’s hunger and satiety sensations, is not in line with the principles of IS.

It appears vital to embed the above-mentioned difficulties with (re-)learning IS and thus IE in a broader societal context: whether for health or ethical reasons, there is a widespread trend of eating according to various kinds of restriction rules. This concerns several types of restrictive dietary practices [66], for instance, regarding specific foods or macronutrients (e.g., low-calorie diet [67] or temporal restrictions (e.g., fasting [68]). Data from a German population-wide consumption study showed that people are dieting more frequently as they age, with around 20% of people over 65 doing so [69]. In light of these trends IE—and more specifically, the training of IS—is facing a challenge. This is also highlighted by a study on IE experiences among British women, where prevailing norms of the diet mentality occurred as barrier for practicing IE [37]. Albeit more implicitly, our findings also suggest a connection of such broader societal norms with the difficulties participants experienced throughout the training. However, there is also an emerging trend towards IE as a counter-movement to the dominant diet culture and related normative discourses [37,70]. It will be the task of future studies to unravel how this affects future cohorts in training IS.

### 4.2. The Role of the Couple Context

Overall, our synthesis pointed at some conflicting results regarding the wish of spousal training involvement. While the quantitative data rather showed that participants did not want to or could not imagine changing the training mode, the FGs highlighted a beneficial effect of spousal training participation. Since the FGs provided more space and time for reflection and encouraged discussions on these issues, it seems plausible that new stimuli and ideas were triggered here. At the same time, mechanisms related to social desirability may have had a stronger impact here, e.g., with respect to the training staff who had designed a couple-based intervention, or to present themselves as a well-attuned couple to the other participants. Indeed, the qualitative data material on this aspect was particularly rich, underlining its credibility despite the contradictory quantitative results. Moreover, the insights gained regarding the beneficial effects are congruent with prior study findings on general dyadic behavior change interventions [46,47,71].

While in the FGs, the involvement of the spouse in the training was considered to be favorable overall, some results also implied that the partner was experienced as an obstacle to adequately practice IS. As an example, one participant described difficulties in concentrating on her own body signals (e.g., with respect to the portion size) in the presence of her partner, because this might have disrupted the social, communicative function of commensality. This emphasizes a balancing act between eating according to hunger and satiety signals and fulfilling the personal needs of closeness and belonging, as has previously been stressed [72,73].

One of our main findings concerned the Partnership Unity (PU) presented by the couples, which manifested itself on various levels—primarily on the level of diet-related everyday practices and (in)congruent daily rhythms and meal structures. According to a previous study, married couples showed (fairly) higher levels of mindful and IE skills as compared to singles. The authors concluded that this was due to couples having more regular daily routines and eating habits as compared to singles [74]. It is well-established that couples tend to share similar taste preferences and dietary practices habits [75,76]. On the one hand, this can be attributed to the phenomenon of “homogamy”, i.e., a similarity in the milieu background and lifestyles from the onset of the relationship [75]. On the other hand, this also results from various negotiation and convergence processes over the course of the relationship [41,75,77].

Two previous studies using data of the NutriAct Family Study (NFS) analyzed such intra-couple dynamics shaping dietary preferences. First, a primarily asymmetrical convergence pattern was found, whereby one partner predominantly adapted the other one’s food preferences [41]. Second, the dominant role of women in shaping older-aged couples’ dietary habits was shown [41,45]. Such convergence dynamics were also evident in the qualitative FGs of the present study. Generally, however, our couples demonstrated a rather symmetrical, conjointly shaped harmonization, specifically with respect to food choice and mealtime routines.

Focusing on meal practices, prior work on intra-couple dynamics highlights jointly-shaped synchronizations that occurred chronologically across different phases [78]. Thereby, synergetic mealtime practices resulted from the last of three synchronization stages. In this stage, previous individual practices and negotiations thereof are blended or combined, amongst others. Given the emphasized PU, our couples may cautiously be attributed to this stage. Besides such convergence processes, various couple biographical aspects (e.g., relationship length, disruptions, and relationship quality) may have been involved in shaping the spouses’ IS- and more general IE-related practices. As previous research suggests, the level of existing IE skills is associated with different relationship types and the perceived relationship quality [38]. Based on our data, we can only assume a relatively high perceived relationship quality, considering how the participants positioned themselves as couples and their PU in the FGs.

### 4.3. Joint Training Execution within Various Daily Routines

There is ample evidence that biographical transitions shape lifestyle behaviors such as dietary practices [8,79]. For our target group, the retirement transition represents such a critical momentum. There are often asynchronous everyday structures and corresponding meal routines determined by everyday working life, as also shown in our FGs. The retirement transition offers an opportunity to (re-)establish more flexible and shared mealtime routines [80]. In the present study, the retirement status of one or both partners also played a role in the joint participation and the establishment of IS and IE practices, which was mostly related to the (dis)similar daily rhythms of the spouses. At the same time, as some of our FG results indicated, even if both partners are in the same phase of life, they may have different daily mealtime routines and only sometimes share their meals, e.g., because they prioritize other (joint) activities. Therefore, retirement may, to some extent, be a facilitating and inhibiting condition for a joint participation in (dietary) couple interventions. In a broader sense, however, it is the similarity of the life phases in general that appears to be decisive, yet further research is needed here.

Broader societal disruptions, such as the COVID-19 pandemic, also play a role in this respect. Findings of an observational study suggest a positive relationship between the pandemic and its lock-down and pronounced levels of mindful eating and IE skills [74]. Our study took place at the time of the COVID-19 lockdown in Germany. This period entailed changes in (shared) daily structures (e.g., working from home) and mealtime routines for many. As implied by some of our FG findings, it may have, hence, created specific facilitating conditions for the couples’ engagement in joint IE practices.

Moreover, the (in)congruent daily rhythms described by our couples can also be framed in terms of broader societal developments. In the context of ongoing individualization processes [81,82], (commensal) traditional day structures are continuously changing. Thus, dietary habits are becoming increasingly flexible, and meals are more frequently eaten alone, rather than in the family context [72,76,83]. In our study, mostly positive meaning and a rather great importance was attached to commensality. However, couples with rather dissimilar daily rhythms described various barriers they faced in the joint training realization. It appears, thus, worthwhile to account for such continually changing everyday realities in future (couple-based) dietary intervention designs. In this way, the practical obstacles to implementing the intervention, as also reported by our couples, could be overcome.

### 4.4. Overlapping Concepts: Interoceptive Sensitivity, Intuitive Eating and Mindfulness

Looking at the IE construct developed by Tribole and Resch [55], our intervention study primarily focused on the improvement of IS and thus the principles “*Honour your hunger*” and “*Feel your fullness*”. Nevertheless, the participants’ discussions also concerned several other principles, such as “*Honour your health-gentle nutrition*”, “*Reject the diet-mentality*”, “*Challenge the food police*” and “*Discover the satisfaction factor*”. Closely related, they also referred to several changes towards more IE-oriented lifestyles and dietary practices as a result of the intervention (e.g., eating in silence and heightened meal anticipation). Such intervention-induced changes were perceived as successful training outcomes, yet went beyond the original objectives of the intervention, and may, thus, be seen as an additional “transfer achievement”. At the same time, the participants also found that the training goals were presented too vaguely or abstract and would have liked more detailed information about the conceptual basis and more specific training instructions. In particular, the desire for feedback and comparability with others (e.g., “*whether we are marching in the same direction*”) is notable here, as this demonstrates insecurities with the concepts of IS and IE and stresses their need for more guidance.

Furthermore, the participants associated the interoception-oriented training with more general mindful eating practices [84] (e.g., eating slowly and focusing on the chewing process) or understood IS and mindfulness as being synonymous. The BS is a mindfulness-based exercise that is not directly linked to the specific interoceptive perception of hunger and satiety cues, but rather to a more generic mindfulness towards one’s own body. In this respect, the link between mindfulness and IS/IE was already drawn in the context of the intervention. Furthermore, other studies also seem to mix up the concepts of IE and mindfulness, as has been critically remarked by Erhardt (2021) [37]. The question, therefore, arises as to the extent to which a strict separation of these concepts is appropriate and useful for IE interventions. Accordingly, it is also questionable in how far the theoretically defined concepts of IS, IE, and mindfulness are indeed distinguishable and distinctively implementable by laypeople. Our findings emphasize these questions, as our participants’ already existing mindfulness orientation seemed to have acted as a facilitator in conducting the training exercises. At the same time, the exercises also stimulated further mindfulness practices, indicating a mixing of the concepts of IS, IE and mindfulness.

### 4.5. Strengths and Limitations

This is the first acceptance study on a novel couple-based intervention program focusing on the (re-)learning of IS skills without the direct goal of weight loss or health-improvement. Our analysis followed a theoretically oriented iterative approach, drawing on the Theoretical Acceptance Framework by Sekhon et al. [49,50]. So far, there was a lack in consented acceptance definitions and respective theory-driven acceptance analyses on behavioral healthcare interventions [49]. Responding to this gap, the TFA is the first framework providing a definitional and, at the same time, analytical framework [50]. Our study is the first to apply the TFA to an intervention acceptance analysis. Using an explorative qualitative convergent synthesis design involving the Pillar Integration Process (PIP) [58], six out of eight TFA-acceptance components emerged as themes. The benefits of MM in behavioral intervention research, amongst others, for generating a rich and comprehensive understanding has been stressed previously [85]. In our MM design, the explorative-inductive analysis process allowed going beyond the TFA acceptance dimensions and providing in-depth insights into various contextual factors.

Since we conducted a pilot trial, we were not able to recruit a representative sample for German adults aged 50 years and above. Furthermore, although we followed a selective recruitment strategy, the IE levels of our intervention participants were comparable to a German community sample. Especially with regard to the training effects, it should be noted that our participants had previously taken part in the NFS and, therefore, were already experienced with diet-related studies. This might reflect the participants’ strong interest and great knowledge about diet-related topics. These aspects might limit the generalizability of our findings. Furthermore, the quantitative measurement of the training evaluation and the intervention acceptance was based on self-constructed items. By the time of the intervention, there was a lack of a validated questionnaires to assess the acceptance of healthcare interventions [50].

With respect to the FGs, there are two major limitations to be addressed: first, cross-case and cross-group comparability was only possible to a limited extent. Due to the COVID-19-restrictions, as well as recruitment problems associated with the pandemic, two of our groups consisted of two people only. Ideally, FGs involve 4-8 individuals, as group sizes may have a crucial effect on interaction dynamics. Moreover, a small group size limits the variety of perspectives, and, at the same time, the discovery of a so-called “collective perspective” [86]. However, our qualitative data material yielded detailed descriptions and showed some contrasting horizons of experiences [87]. Second, specific FG dynamics may occurred due to mechanisms related to social desirability and (self-)positioning as a couple, i.e., the ways in which the individuals and couples portrayed themselves towards the other participants as well as the researcher [88]. In our study, this might have been specifically the case with respect to the couples’ representations of their PU, which were emphasized in various ways and to varying degrees. To address the above-mentioned shortcomings, future studies are needed that use qualitative theoretical sampling in further investigating, amongst others, intra-couple dynamics involved in IE-related practices.

### 4.6. Implications for Future Interventions

Against the background of the intervention acceptance and contextual factors discussed above, several conclusions can be drawn for future interventions. Most importantly, the participants expressed a desire for closer guidance and feedback during the intervention, as well as a personalized evaluation after the study. It seems, hence, vital to promote a better understanding and a more thorough implementation, especially of the HU and SA exercises. First, it might be beneficial to provide more detailed information and conceptual definitions regarding the concepts IS and IE, as well as to explain the intentions of each exercise more explicitly in order to help the participants to monitor their own learning progress. Second, a regular involvement and (personal or digital) exchange with the study staff and also the opportunity to share experiences with other participants could be fruitful. Here, a(n) (online) group setting (e.g., group videoconferencing) appears to be effective [25,89], which, for example, involves an exchange forum during the respective training weeks.

The participants’ wish for interaction may have been related to the web-based study format. Previous studies have challenged web-based behavioral intervention designs and concluded face-to-face-designs to be more effective [25]. This may also be true for our intervention, because older participants might need greater support than younger participants regarding study participation and the conduction of an online training [90]. However, not only during COVID-19 times, web-based interventions may be particularly feasible and practicable, since they are readily available and have rather low access barriers [91,92].

Overall, our findings support benefits of dietary couple-based interventions, which have also been suggested by previous research [47,93]. Regarding the couple-related aspects discussed above, it appears worthwhile for future diet-related interventions to target couples who share similar daily routines or aim to do so. Since different life phases and daily rhythms can hinder a regular joint training participation, the question arises as to in which cases and under which circumstances a couple-based intervention is appropriate and expedient. In a previous intervention combining IE and mindfulness for the treatment of maladaptive eating behaviors, the integration of intervention programs into the workplace or other (institutionalized) settings was emphasized as a successful strategy [94]. How such an approach can be meaningfully applied to the target group of people 50+ and especially to couples is the task of future studies.

Finally, our sample showed IE levels comparable to adults aged 50+ in a German community sample [11] and a general pronounced mindfulness and health orientation. Previous studies have discussed IE as a “privileged approach” [95] and shown “food secure adults” [26] to score higher in IE skills. Hence, future studies on population groups with more diverse and precarious socio-economic backgrounds as well as lower IE skills are needed and a respective tailoring of IS/IE interventions seems essential.

## 5. Conclusions

Intuitive Eating (IE)—an adaptive eating behavior characterized by an attunement to one’s hunger and satiety signals—has gained scientific interest as a critical response to dieting trends. Training interoceptive sensitivity (IS) might be a first step in effectively promoting IE. The couple context plays an important role in (re-)learning and changing dietary practices, including IE. The benefits of couple-based health interventions in general have been demonstrated, but no such studies have yet been conducted in relation to IE. Against this background, a web-based dyadic experimental interoception-based pilot randomized-controlled trial (RCT) was conducted to increase IS—and, thus, IE—among couples aged 50 years and older. The training consisted of three exercises involving a more general mindful-oriented Body Scan (BS) and two exercises specifically concerning IS towards hunger (HU) and satiety (SA).

The present acceptance study was the first to exploratively analyze how couples accept a dyadic interoception-based training program to increase IS and, to explore the role the couple context in the experience and conduct of a joint vs. single training execution. In a mixed-methods convergent synthesis design, findings of a quantitative survey (*n* = 68 couples) and of qualitative focus groups (FGs) (*n* = 4 couples with 12 individuals) were synthesized using the Pillar Integration Process (PIP). The synthesis process was oriented towards the Theoretical Framework of Acceptance (TFA).

Overall, a fairly moderate general acceptance emerged, e.g., with respect to its feasibility and low burden. A hierarchical gradient was shown in favor of the BS, which was associated with benefits beyond the training’s intention, such as an improved sleep quality. Amongst others, barriers concerned a perceived lack of the exercises’ usefulness and a limited understanding of the training’s intended purpose. The participants expressed a wish for regular feedback and exchange with the study stuff and other participants. A rather beneficial effect of having the partner involved in the training execution was demonstrated. Thereby, a jointly established Partnership Unity regarding previously harmonized diet-related practices and shared daily routines (e.g., mealtime structures) appeared as constructive pre-condition for a joint training success.

Our study highlights the potential and implications of a web-based training of IS in a couple context among adults aged 50+. Further studies are needed that address different population groups. Future interventions should take into account the couple context and focus on a regular exchange with other participants and a closer guidance by the study staff to promote a better understanding of the processes and goals of IS and IE.

## Figures and Tables

**Table 1:** QUAN sample characteristics.

		Group 1		Group 2		Group 3	
Couples (*n*)		24		22		22	
		G1-I	G1-P	G2-I	G2-P	G3-I	G3-P
Group size (*n*)		24	23	22	22	22	21
Gender							
	Women (*n*)	10	14	10	12	14	8
	Men (*n*)	14	9	12	10	8	13
Age (years)							
	*M* (*SD*)	66.0 (4.2)	67.2 (6.1)	67.7 (4.9)	67.4 (6.0)	67.8 (6.4)	68.5 (6.2)
	Min–Max	56–74	53–82	60–77	56–77	58–85	57–80
BMI (kg/m^2^) ^1^							
	*M* (*SD*)	27.8 (4.8)	26.4 (4.9)	27.3 (3.6)	26.0 (3.1)	25.5 (3.1)	23.5 (3.3)
	Min–Max	19.0–39.0	20.8–42.2	20.6–35.7	22.2–34.1	20.6–32.5	17.6–28.4

Note. ^1^ BMI was calculated from height and weight (BMI = weight (kg)/height (m) [64]. BMI values for index persons were based on laboratory measurements of height and weight, while BMI values for partners were based on self-reported height and weight.

## Data Availability

As this study is part of the ongoing NutriAct Family Study, access to data will be arranged on a reasonable request and with the permission of all collaboration partners.

## Supplementary Materials

The following supporting information can be downloaded at: https://www.mdpi.com/article/10.3390/nu16121949/s1. Multimedia Supplementary S1: Synthesis Table according to the Pillar Integration Process.
