# Supplementary material for: Practicing Interoceptive Sensitivity as a Couple: A Mixed-Methods Acceptance Analysis of a Dyadic vs. Single Pilot Randomized Controlled Trial
Source: Nutrients. 2024 Jun 19;16(12):1949. doi: 10.3390/nu16121949 (PMC11206497; doi:10.3390/nu16121949)
Supplement: Supplementary file 1 [file nutrients-16-01949-s001.zip › nutrients-3015326-supplementary.pdf]

Synthesis Table according to the Pillar Integration Process (Johnson et al. 2017)

QUANT Data	QUANT Categories	Pillar building Themes	QUAL Categories	QUAL Data
**OVERALL INTERVENTION**				
**RV_1: Perception of improvement of hunger and satiety signals**<br>*M* (*SD*) = 3.16 (1.44) (*n* = 55)<br>• 1 (not at all): 16.4 % (*n* = 9)<br>• 2: 21.8 % (*n* = 12)<br>• 3: 14.5 % (*n* = 8)<br>• 4: 23.6 % (*n* = 13)<br>• 5: 23.6 % (*n* = 13)<br>• 6 (very much): 0 % (*n* = 0)	**Medium subjectively perceived training success**	**<u>PERCEIVED EFFECTIVENESS</u>**	**Theoretical knowledge gain**	*„Well, I learned quite a lot from this, I have to say" (FG couples, 14).*
**ARV_5: Changes in everyday life**<br>• No: 78.7 % (*n* = 48)<br>• Yes: 18.8 % *(n* = 13) (of which 46.2 % reported increased levels of IS)	**Few changes in everyday life, reported changes:**<br>- Increased ability for IS/ awareness for body signals		**Practical knowledge gain regarding dietary practices:**<br>- Meal setting<br>- Eating pace<br>- Portion size	*„Now [after the intervention], we have started to eat in silence; largely, not always" (FG couples, 30).*<br>*„I used to be quite the <u>fast eater</u>. [...] And after the study, I started to consciously eat more slowly and <u>chew a lot</u>" (FG couples, 31).*<br>*„Yeah, but [now] I notice I am full, there was too much on my plate again [...]. But next time it might be a little less [smaller portion size]" (FG mixed, 189).*
**Mean_IES-2**<br>M_{total} (*SD*) = 3.64 (0.46) (*n* = 128)<br>$M_{index\ persons}$ (*SD*) = 3.49 (0.46) (*n* = 66)<br>$M_{partners}$ (*SD*) = 3.81 (0.38) (*n* = 62)<br>• $M_{G1\text{-}I}$ (*SD*) = 3.57 (0.46) (*n* = 24)<br>• $M_{G1\text{-}P}$ (*SD*) = 3.84 (0.39) (*n* = 21)	**Rather high levels of pre-existing IE skills**		**Increased ability for IS/ awareness for body signals**	*"For me, the most import thing from the study was to pay even better attention to these satiety signals perhaps, or even in general, what my body is telling me" (FG couples, 23)".*<br>*"So, I consider the word 'mindfulness' as very important and I think, <u>for me</u>, the greatest <u>benefit</u> [from the intervention] was that we just*

• $M_{G2\text{-}I}$ (*SD*) = 3.43 (0.38) (*n* = 21)<br>• $M_{G2\text{-}P}$ (*SD*) = 3.75 (0.42) (*n* = 22)<br>• $M_{G3\text{-}I}$ (*SD*) = 3.45 (0.55) (*n* = 21)<br>• $M_{G3\text{-}P}$ (*SD*) = 3.85 (0.35) (*n* = 19)				*payed more attention again to the body signals" (FG couples, 224).*
	n.a.	**INTERVENTION COHERENCE**	**Understanding of the intervention's intended benefit**	*„So, in principle, I realized what it [the exercise] was supposed to do; that you take a moment before eating and contemplate and reflect on yourself and listen to your body, whether and how hungry you actually are" (FG men, 123).*<br><br>*„And then I thought to myself: yes, of course. When it comes to eating, perhaps that also plays @a decisive role@, er, how much you eat, what you eat…that you are guided to pay attention to your bodily signals" (FG couples, 223).*
**Comparison of Training Exercises**				
**Mean_TES_1: Overall evaluation of each exercise**<br>• BS: *M* (*SD*) = 4.46 (0.54) *(n* = 44)<br>• HU: *M* (*SD*) = 4.12 (0.67) *(n* = 44)<br>• SA: *M* (*SD*) = 4.23 (0.63) *(n* = 44)<br>**Friedman's ANOVA**<br>X^2 (2) = 11.93, *p* = .003<br>Post-hoc comparison: $M_{BS} > M_{HU}$	**Hierarchical evaluation of exercises in favor of the BS**	**AFFECTIVE ATTITUDES**	**Preference of BS over HU and SA exercises**	*„That one, I liked really much and even my husband enjoyed it. But the other two exercises, they gave us absolutely nothing" (FG women, 55).*
	**Overall Acceptance**			
**ARV_2: Recommendation of training**<br>*M* (*SD*) = 3.87 (1.38) (*n* = 55)<br>• 1 (not at all): 3.6 % (*n* = 2)<br>• 2: 16.4 % (*n* = 9)<br>• 3: 16.4 % (*n* = 9)<br>• 4: 29.1 % (*n* = 16)	**Majority would recommend the intervention to others**	**GENERAL ACCEPTABILITY**	**Recommendation**	*"Everyone who is not taking part in this study should also do this for themselves. Well, perhaps more [information on this] should be made public, so that people simply take this concept of saturation into account, right?" (FG mixed, 797).*<br><br>*"That kind of means that young people should participate in this kind of dietary study, right? Because I know that currently many, well let me*

• 5: 21.8 % (*n* = 12)<br>• 6 (absolutely): 12.7 % (*n* = 7)				*say overweight people, are really, really young. So that might also be an approach: to invite the younger folks to this kind of intervention to make them realize how important this is, especially for later life. Because if they are already that overdimensioned now, how will this be in old age? That is really important, right?" (FG mixed, 36).*

Synthesis Table according to the Pillar Integration Process (Johnson et al. 2017)

<table>
<tr><th colspan="5">BODYSCAN (BS)</th></tr>
<tr>
<td rowspan="2">TES_1_6: Usefulness for everyday life
• BS: M (SD) = 3.87 (1.12) (n = 45)
• HU: M (SD) = 3.29 (1.12) (n = 45)
• SA: M (SD) = 3.09 (1.14) (n = 44)
Friedman’s ANOVA:
X^2 (2) = 16.29, p < .001
Post-hoc comparison: $M_{BS} > M_{SA}$</td>
<td rowspan="2">Rather pronounced usefulness for everyday life

(significantly higher perceived usefulness for everyday life for BS compared to SA)</td>
<td rowspan="2"><u>PERCEIVED EFFECTIVENESS</u>
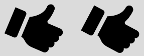</td>
<td>Improved ability for IS/ awareness for body signals

- relaxation, improved sleep quality</td>
<td>„It was the first time I <u>consciously</u> felt my body in every corner, right? And then I lay down for another moment and afterwards I got up, let‘s say, as light as a feather; I was calm, also calm inside, and found it really fascinating what it does to your mind" (FG Mixed, 157).

„I even felt like I could sleep better. Yes, exactly. You've somehow brought your body down” (FG couples, 187).</td>
</tr>
<tr>
<td>No perceived changes</td>
<td>“Well, I am not aware that this has changed anything for me” (FG men, 110). „This relaxation exercise is okay, but I neither can really tell you whether it would or has had an influence on my eating behavior” (FG women, 103).</td>
</tr>
</table>

**TES_1_1: Exertion**<br>• BS: *M* (*SD*) = 4.71 (0.70) (*n* = 45)<br>• HU: *M* (*SD*) = 4.51 (0.82) (*n* = 45)<br>• SA: *M* (*SD*) = 4.70 (0.59) (*n* = 44)<br>**Friedman's ANOVA: n.s.**<br>X^2 (2) = 4.154, *p* = .125<br><br>**TES_1_2: Easy to follow**<br>• BS: *M* (*SD*) = 4.67 (0.64) (*n* = 45)<br>• HU: *M* (*SD*) = 4.67 (0.56) (*n* = 45)<br>• SA: *M* (*SD*) = 4.79 (0.47) (*n* = 43)<br>**Friedman's ANOVA: n.s.**<br>X^2 (2) = 2.130, *p* = .345<br><br>**TES_1_3: Comprehensibility**<br>• BS: *M* (*SD*) = 4.86 (0.35) (*n* = 44)<br>• HU: *M* (*SD*) = 4.72 (0.50) (*n* = 43)<br>• SA: *M* (*SD*) = 4.80 (0.51) (*n* = 44)<br>**Friedman's ANOVA: n.s.**<br>X^2 (2) = 2.882, *p* = .237	**Low levels of effort required**<br><br>**High levels of comprehensibility**	**<u>BURDEN</u>**<br>**&**<br>**<u>SELF-EFFICACY</u>**	**Easy realization**<br><br>- Prior experiences<br>**>> Facilitator**	*„The scan later became automatic. Then, you didn't even need to hear the voice, you were already going through your body all by yourself" (FG couple, 81).*<br><br>*„Well, I <u>was already able</u> to do this exercise because I always do it, so I had no difficulties with it" (FG mixed, 183).*
**TES_1_4: Pleasantness**<br>• BS: *M* (*SD*) = 4.56 (0.79) (*n* = 45)<br>• HU: *M* (*SD*) = 4.05 (0.91) (*n* = 44)<br>• SA: *M* (*SD*) = 4.32 (0.83) (*n* = 44)<br>**Friedman's ANOVA:**<br>X^2 (2) = 11.878, *p* = .003, but no significant post-hoc comparison	**High levels of pleasantness**	**<u>AFFECTIVE ATTITUDES</u>**	**Positive emotions**<br><br>- Enjoyability<br><br>- Pleasantness	*„[…] the Body Scan was excellent for me, as I'm a bit of a fidgety type anyway...but I managed it well at the time [(of the intervention)]"<br>(FG mixed, 173).*<br><br>*„But he was <u>really pleased by</u> the Body Scan. // Dagmar: nice! // At the beginning he said 'that's hocus-pocus' or something like that and I said 'why don't you try it first', yeah? And*

**TES_1_5: Liking**<br>• BS: *M* (*SD*) = 4.23 (1.08) (*n* = 44)<br>• HU: *M (SD)* = 3.61 (1.17) (*n* = 44)<br>• SA: *M* (*SD*) = 3.75 (1.28) (*n* = 44)<br>**Friedman's ANOVA:**<br>X^2 (2) = 10.938, *p* = .004<br>Post-hoc comparison: $M_{BS} > M_{HU}$	**High levels of liking (*significantly higher for BS compared to HU*)**			*[(afterwards)] he then […] that was really pleasant!'" (FG women, 68)*
			**Negative emotions**<br>- Annoyance<br>- Disappointment	*„That gets on my nerves at some point and it makes me fidgety" (FG men, 92)*<br><br>*"I thought something <u>special</u> has to happen here. // Dirk: A sensation. // Christian: (laughs). Well, it didn't. The sensation didn't happen. And I am still waiting for it today (laughs), indeed." (FG men, 106-108)*
**(Dis)continuation of BS**				
**ES_2: Intended BS continuation after intervention**<br>• No: 22.2 % (*n* = 10)<br>• Yes: 77.8 % (*n* = 35)<br><br>**ARV_6: Actual continuation of exercises**<br>• 1 (not at all): 54.1 % (*n* = 33)<br>• 2 (1x): 4.9 % (*n* = 3)<br>• 3 (2-3x): 13.1 % (*n* = 8)<br>• 4 (1x a week): 13.1 % (*n* = 8)<br>• 5 (Several times a week): 6.6 % (*n* = 4)<br>• 6 (1x a day): 8.2 % (*n* = 5)	**Continuation of BS intended by majority**<br><br>**Discontinuation** (Not at all: 54 %) **vs. Continuation to varying degrees** (Sporadically: 18 %, Regularly: 20 %, Daily: 8 %) (of all exercises)	**<u>GENERAL ACCEPTABILITY</u>**<br>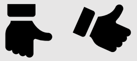	>> many participants: **Regular continuation**<br><br>**Irregular/ if needed**<br>>> few participants: **Discontinuation, reasons:**	*„I have really kept this one going continuously" (FG women, 51). „Or if you <u>cannot</u> sleep […]. It calms you down, so it's sleep-inducing, if you can't sleep" (FG couples, 202). „Well, I neither do it regularly. I somewhat do it as needed" (FG couples, 201).*<br><br>*"Or I'm […] already that perfect, that it can't change for the better, right? […] (laughs)" (FG men, 113).*<br><br>*„Well, I can-can't say that I've somehow erm have noticed any lasting effect" (FG men, 122).*

**Mean_IES-2**<br>M_{total} (*SD*) = 3.64 (0.46) (*n* = 128)<br>$M_{index\ persons}$ (*SD*) = 3.49 (0.46) (*n* = 66)<br>$M_{partners}$ (*SD*) = 3.81 (0.38) (*n* = 62)<br>• M_{G1-I} (*SD*) = 3.57 (0.46) (*n* = 24)<br>• M_{G1-P} (*SD*) = 3.84 (0.39) (*n* = 21)<br>• M_{G2-I} (*SD*) = 3.43 (0.38) (*n* = 21)<br>• M_{G2-P} (*SD*) = 3.75 (0.42) (*n* = 22)<br>• M_{G3-I} (*SD*) = 3.45 (0.55) (*n* = 21)<br>• M_{G3-P} (*SD*) = 3.85 (0.35) (*n* = 19)	**Rather high pre-existing IE skills**		- Pre-existing abilities<br>- No understanding of purpose<br>- No added value<br>- No personal necessity<br>(High) expectations<br>**>> Barrier**	*„By the way, he practices Quigong. And erm he said: 'Nah, I don't need this kind of relaxation exercise'. He already feels very relaxed by the Quigong" (FG women, 67).*<br>*„Well, I had this kind of […] indefinable underlying expectation, I thought something special must yet happen here" (FG men, 106).*

**HUNGER EXERCISE (HU)**				
**TES_1_6: Usefulness for everyday life**<br>• BS: *M* (*SD*) = 3.87 (1.12) (*n* = 45)<br>• HU: *M* (*SD*) = 3.29 (1.12) (*n* = 45)<br>• SA: *M* (*SD*) = 3.09 (1.14) (*n* = 44)<br>**Friedman's ANOVA**<br>X^2 (2) = 16.29, $p < .001$<br>Post-hoc comparison: $M_{BS} > M_{SA}$	**Moderately perceived usefulness for everyday life**	**<u>PERCEIVED EFFECTIVENESS</u>**<br>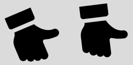	**Knowledge gain**	*"I was curious to see if there were any new aspects in addition to what we were already doing. Those hunger and satiety exercises were new aspects for me. And I'm grateful for that, indeed."* (FG couples, 89).
**Mean_IES-2**<br>M_{total} (SD) = 3.64 (0.46) (*n* = 128)<br>$M_{index\ persons}$ (SD) = 3.49 (0.46) (*n* = 66)<br>$M_{partners}$ (SD) = 3.81 (0.38) (*n* = 62)<br>• M_{G1-I} (SD) = 3.57 (0.46) (*n* = 24)<br>• M_{G1-P} (SD) = 3.84 (0.39) (*n* = 21)<br>• M_{G2-I} (SD) = 3.43 (0.38) (*n* = 21)<br>• M_{G2-P} (SD) = 3.75 (0.42) (*n* = 22)<br>• M_{G3-I} (SD) = 3.45 (0.55) (*n* = 21)<br>• M_{G3-P} (SD) = 3.85 (0.35) (*n* = 19)	**Rather high levels of pre-existing IE skills**		**Improved ability for IS/ awareness for body signals**	*„[…] in <u>retrospect</u>, in the time since [the intervention], I have to say I've become more aware of it. I realise better when I'm hungry and when I'm full. I've become more aware of this feeling. I used to notice it less"* *(FG mixed, 185).*
			**No (lasting) training effects**	*"I just go through the (.) - the digestive organs a bit and feel what I'm sensing inside myself before eating. And I'm of the opinion that I can really, uh- so if we don't turn on the radio or something, which we don't do any more, uh that I can then (.) also maintain a bit of mindfulness, uh not always, but more and more often; and sometimes, I don't eat faster than you [(partner)], but finish at the same time - but not always"* *(FG Couples, 90).*<br><br>*„I also took part in these [other exercises], but without any lasting effects."* (FG men, 87).<br><br>*„I can't say that <u>anything</u> has changed as a result. <u>Not at all.</u> And that's why I questioned myself and said: 'what's the point if that doesn't lead to anything at all"* *(FG women, 131).*

**TES_1_4: Pleasantness**<br>• BS: *M* (*SD*) = 4.56 (0.79) (*n* = 45)<br>• HU: *M* (*SD*) = 4.05 (0.91) (*n* = 44)<br>• SA: *M* (*SD*) = 4.32 (0.83) (*n* = 44)<br>**Friedman's ANOVA:**<br>X^2 (2) = 11.878, *p* = .003, but no significant post-hoc comparison	**High levels of pleasantness**	**AFFECTIVE ATTITUDES**	**Positive emotions**<br>- Heightened meal anticipation<br>- Curiosity<br>- Gratitude for knowledge gain	*„At the end [of the HU exercise] I always told myself: 'So, now you can have a delicious dinner!'. […] And then I was really looking forward to eat something and I really actively went upstairs and said: 'Oh, now you are allowed to eat something really nice!'" (FG mixed, 484). „I was curious to see if there were any new aspects in addition to what we were already doing. Those hunger and satiety exercises were new aspect for me. And I'm grateful for that, indeed." (FG couples, 89).*
**TES_1_5: Liking**<br>• BS: *M* (*SD*) = 4.23 (1.08) (*n* = 44)<br>• HU: *M* (*SD*) = 3.61 (1.17) (*n* = 44)<br>• SA: *M* (*SD*) = 3.75 (1.28) (*n* = 44)<br>**Friedman's ANOVA:**<br>X^2 (2) = 10.938, *p* = .004<br>Post-hoc comparison: $M_{BS} > M_{HU}$	**Rather high levels of liking**<br>**(*significantly lower levels of liking for HU compared to BS*)**		**Negative emotions**<br>- Annoyance >> needless/ redundant<br>-<br>- Anger >> illogical, senseless content	*„The hunger exercise sometimes annoyed me [laughs], because I was already hungry and then [laughs] [had to do] a hunger exercise on top of that" (FG couples, 79)".*<br>*„But this hunger exercise and also the satiety exercise, those dove me up the wall, because the questions asked were complete nonsense in my point of view. Like, 'were do I feel hunger'. […] I feel it in my stomach and in the head, sure. So, in the digestive tract I feel hunger. And satiety, too. But I don't feel it in my hands and feet – so, these were completely illogical questions [laughs]. Practically every time these questions came – It was over. That's when I got annoyed" (FG men, 92).*

**TES_1_1: Exertion**<br>• BS: *M* (*SD*) = 4.71 (0.70) (*n* = 45)<br>• HU: *M* (*SD*) = 4.51 (0.82) (*n* = 45)<br>• SA: *M* (*SD*) = 4.70 (0.59) (*n* = 44)<br>**Friedman's ANOVA: n.s.**<br>X^2 (2) = 4.154, *p* = .125<br><br>**TES_1_2: Easy to follow**<br>• BS: *M* (*SD*) = 4.67 (0.64) (*n* = 45)<br>• HU: *M* (*SD*) = 4.67 (0.56) (*n* = 45)<br>• SA: *M* (*SD*) = 4.79 (0.47) (*n* = 43)<br>**Friedman's ANOVA: n.s.**<br>X^2 (2) = 2.130, *p* = .345<br><br>**TES_1_7: Easy to concentrate on**<br>• BS: *M* (*SD*) = 4.13 (0.99) (*n* = 45)<br>• HU: *M* (*SD*) = 3.89 (1.09) (*n* = 45)<br>• SA: *M* (*SD*) = 4.16 (1.14) (*n* = 44)<br>**Friedman's ANOVA: n.s.**<br>X^2 (2) = 3.976, *p* = .137	**Low levels of effort required**<br><br>**Rather low levels of concentration required**	**BURDEN & SELF-EFFICACY**<br>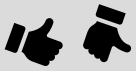	**Difficulties in realization**	***„So, for me, the being seated was a problem, yeah? This- I couldn't, let's say, concentrate as much as when you lie down really comfortably. […] For some reason, I couldn't get to notice my hunger feeling, right? So that's why the exercise was difficult for me […]. When I had finished the five times I was glad, right? Because it was exhausting" (FG mixed, 470).***<br><br>***„Well, you first had to get used to it, to become familiar with it. But it got better over time, then you could- you managed it better, that was my experience" (FG mixed, 469).***

**(Dis)continuation of HU**				
**TES_2: Intended HU continuation after intervention**<br>• No: 70.5 % (*n* = 31)<br>• Yes: 29.5 % (*n* = 13)<br><br>**ARV_6: Actual continuation of exercises**<br>• 1 (not at all): 54.1 % (*n* = 33)<br>• 2 (1x): 4.9 % (*n* = 3)<br>• 3 (2-3x): 13.1 % (*n* = 8)<br>• 4 (1x a week): 13.1 % (*n* = 8)<br>• 5 (Several times a week): 6.6 % (*n* = 4)<br>• 6 (1x a day): 8.2 % (*n* = 5)<br><br>**Mean_IES-2**<br>M_{total} (*SD*) = 3.64 (0.46) (*n* = 128)<br>$M_{index\ persons}$ (*SD*) = 3.49 (0.46) (*n* = 66)<br>$M_{partners}$ (*SD*) = 3.81 (0.38) (*n* = 62)<br>• $M_{G1\text{-}I}$ (*SD*) = 3.57 (0.46) (*n* = 24)<br>• $M_{G1\text{-}P}$ (*SD*) = 3.84 (0.39) (*n* = 21)<br>• $M_{G2\text{-}I}$ (*SD*) = 3.43 (0.38) (*n* = 21)<br>• $M_{G2\text{-}P}$ (*SD*) = 3.75 (0.42) (*n* = 22)<br>• $M_{G3\text{-}I}$ (*SD*) = 3.45 (0.55) (*n* = 21)<br>• $M_{G3.P}$ (*SD*) = 3.85 (0.35) (*n* = 19)<br><br>**Mean_TES_1: Overall evaluation of each exercise**<br>• BS: *M* (*SD*) = 4.46 (0.54) (*n* = 44)<br>• HU: *M* (*SD*) = 4.12 (0.67) (*n* = 44)	**No continuation of HU intended by majority**<br><br>**Discontinuation** (Not at all: 54 %) **vs. Continuation to varying degrees** (Sporadically: 18 %, Regularly: 20 %, Daily: 8 %) (of all exercises)<br><br>**Rather high levels of pre-existing IE skills**	**<u>GENERAL ACCEPTABILITY</u>**<br>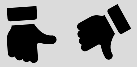	>> very few participants: **Continuation (in part)**<br><br>>> many participants: **Discontinuation, reasons:**<br><br>- No added value<br><br>- Lack of purpose<br><br>- Lack of time **>> Barrier**<br><br>- No sensation of hunger **>> Barrier**	*„And the hunger exercise: we still keep doing it before every breakfast. We haven't managed to repeat it before lunch yet (laughs)" (FG couples, 89).*<br><br>*„And I didn't pursue that any longer then [after the intervention]. Well, I did with the first exercise, but I didn't for the others" (FG women, 60). „To this day, I still don't really see the (deep) meaning of it" (FG men, 91).*<br><br>*„I wonder if maybe you should have extended it as a self-exercise, you know? But I didn't do it, because I have to say that I'm always a bit short on time" (FG men, 134). „Well, I have to add: Just like you [(other participants)] don't know the feeling of satiety, I don't really know the feeling of hunger either" (FG women, 128)*

• SA: *M* (*SD*) = 4.23 (0.63) (*n* = 44)<br>**Friedman's ANOVA**<br>X^2 (2) = 11.93, *p* = .003<br>Post-hoc comparison: $M_{BS} > M_{HU}$				

Synthesis Table according to the Pillar Integration Process (Johnson et al. 2017)

SATIETY EXERCISE (SA)				
**TES_1_6: Usefulness for everyday life**<br>• BS: *M* (*SD*) = 3.87 (1.12) (*n* = 45)<br>• HU: *M* (*SD*)) = 3.29 (1.12) (*n* = 45)<br>• SA: *M* (*SD*) = 3.09 (1.14) (*n* = 44)<br>**Friedman's ANOVA**<br>X^2 (2) = 16.29, *p* < .001<br>Post-hoc comparison: $M_{BS} > M_{SA}$	**Moderately perceived usefulness for everyday life**<br><br>**(*significantly lower perceived usefulness for everyday life for SA compared to BS*)**	**<u>PERCEIVED EFFECTIVENESS</u>**<br>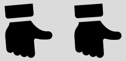	**Increased ability for IS/awareness of body signals**<br><br>- On (pre)reflexive levels	*„So, the feeling of satiety was really the most quiet one for me [at the beginning of the intervention]. […] And this has become a little bit more pronounced" (FG couples, 278).*<br><br>*„So, I […] haven't kept repeating it, but in the back of my head I always wonder: 'Have you possibly eaten <u>too much</u> or how <u>full</u> do you feel?'" (FG couples, 89).*
**Mean_IES-2**<br>M_{total} (*SD*) = 3.64 (0.46) (*n* = 128)<br>$M_{index\ persons}$ (*SD*) = 3.49 (0.46) (*n* = 66)<br>$M_{partners}$ (*SD*) = 3.81 (0.38) (*n* = 62)<br>• M_{G1-I} (*SD*) = 3.57 (0.46) (*n* = 24)<br>• M_{G1-P} (*SD*) = 3.84 (0.39) (*n* = 21)<br>• M_{G2-I} (*SD*) = 3.43 (0.38) (*n* = 21)<br>• M_{G2-P} (*SD*) = 3.75 (0.42) (*n* = 22)<br>• M_{G3-I} (*SD*) = 3.45 (0.55) (*n* = 21)<br>• M_{G3-P} (*SD*) = 3.85 (0.35) (*n* = 19)	**Rather high levels of pre-existing IE skills**		**Restrictive / non-interoceptive eating**	*„What the study did for me: It raised awareness for the concept of satiety. Because I- even though I don't notice if I am already full at that moment, I might notice it minutes later, but by then, I've already had several other forks or spoons. It [now] has rather become a matter of rationality. The concept of satiety has triggered a bit more reason in me, for now I say: 'No, that's enough. You might not be really full yet, but that's enough, stop it.' Right? That's what my consciousness tells me" (FG mixed, 813).*

**Mean_TES_1: Overall evaluation of each exercise**<br>• BS: *M* (*SD*) = 4.46 (0.54) (*n* = 44)<br>• HU: *M* (*SD*) = 4.12 (0.67) (*n* = 44)<br>• SA: *M* (*SD*) = 4.23 (0.63) (*n* = 44)<br>**Friedman's ANOVA**<br>X^2 (2) = 11.93, *p* = .003<br>Post-hoc comparison: $M_{BS} > M_{HU}$			**No perceived changes**<br>- Pre-existing decelerated/ mindful eating practices **>> Facilitator**	*„So, erm, my- my perception of satiety and the feeling of hunger has not improved as a result" (FG men, 94).*<br><br>*„So, for us [couple], the meal is a ceremony in a certain sense, right? Not that we make a big fuss about it, but it all happens in a calm manner. We chew thoroughly without counting the amount of chewing actions per bite. But yeah, it all happens in peace" (FG men, 124).*<br><br>*„I would actually like to emphasize [that] the- the exercise itself didn't give me anything, because I already live according to this pattern anyway, right?" (FG men, 187).*
**TES_1_1: Exertion**<br>• BS: *M* (*SD*) = 4.71 (0.70) (*n* = 45)<br>• HU: *M* (*SD*) = 4.51 (0.82) (*n* = 45)<br>• SA: *M* (*SD*) = 4.70 (0.59) (*n* = 44)<br>**Friedman's ANOVA: n.s.**<br>X^2 (2) = 4.154, *p* = .125<br><br>**TES_1_2: Easy to follow**<br>• BS: *M* (*SD*) = 4.67 (0.64) (*n* = 45)<br>• HU: *M* (*SD*) = 4.67 (0.56) (*n* = 45)<br>• SA: *M* (*SD*) = 4.79 (0.47) (*n* = 43)<br>**Friedman's ANOVA: n.s.**<br>X^2 (2) = 2.130, *p* = .345<br><br>**TES_1_3: Comprehensibility**<br>• BS: *M* (*SD*) = 4.86 (0.35) (*n* = 44)<br>• HU: *M* (*SD*) = 4.72 (0.50) (*n* = 43)	**Low levels of effort required**<br><br>**High levels of comprehensibility**	**<u>AFFECTIVE ATTITUDES & SELF-EFFICACY</u>**	**Difficulties with exercise**	*„The same goes for the satiety exercise. It was exhausting because I didn't realize if I was full or not, what is that feeling?" (FG mixed, 470).*<br><br>*„[…] and maybe I had more of a problem with the satiety exercise than with the hunger exercise. So, hunger is easier to identify for me than satiety" (FG couples, 288)".*

• SA: *M* (*SD*) = 4.80 (0.51) (*n* = 44)<br>**Friedman's ANOVA: n.s.**<br>X^2 (2) = 2.882, *p* = .237				
**(Dis)Continuation of SA Exercise**				
**TES_2: Intended SA continuation after intervention**<br>• No: 63.6 % (*n* = 28)<br>• Yes: 36.4 % *(n* = 16)<br><br>**ARV_6: Actual continuation of exercises**<br>• 1 (not at all): 54.1 % (*n* = 33)<br>• 2 (1x): 4.9 % (*n* = 3)<br>• 3 (2-3x): 13.1 % (*n* = 8)<br>• 4 (1x a week): 13.1 % (*n* = 8)<br>• 5 (several times a week): 6.6 % (*n* = 4)<br>• 6 (1x a day): 8.2 % (*n* = 5)	**No continuation of SA intended by majority**<br><br>**Discontinuation** (Not at all: 54 %) **vs. Continuation to varying degrees** (Sporadically: 18 %, Regularly: 20 %, Daily: 8 %) (of all exercises)	<u>**GENERAL ACCEPTABILITY**</u><br>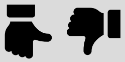	**Discontinuation**<br><br>- Pre-existing abilities >> no necessity<br><br>- No added value<br><br>- Dietary habits shaped by the socialization context **>> Barrier**	*„But, to be honest, I don't really do the satiety exercise" (FG couples, 288). „So maybe I don't necessarily <u>need</u> it" (FG couples, 90).*<br><br>*„I wouldn't say that it didn't give my <u>anything</u> (at all), but I was just already eating more consciously before [the training]" (FG couples, 284).*<br><br>*„I was actually raised to always eat up, you know? (laughs). That's why I always finish my plate" (FG mixed, 213).*

Note. Thumb symbols indicate positive and negative experiences/attitudes/evaluations; G1-I = Index person group 1, G1-P = partner group 1, G2-I = Index person group 2, G2-P = partner group 2, G3-I = Index person group 3, G3-P = partner group 3; IE = Intuitive Eating; IS = Interoceptive Sensitivity; BS=Body Scan, HU = hunger exercise, SA = satiety exercise; the names in the QUAL citations are pseudonyms

Synthesis Table according to the Pillar Integration Process (Johnson et al. 2017)

QUANT Data	QUANT Categories	Pillar Building Themes	QUAL Categories	QUAL Codes
**ARV_3: Preference for training**<br>• 1 (with partner) = 67.6 % (*n* = 25)<br>• 2 (alone) = 32.4 % (*n* = 12)<br><br>**ARV_4: Preference for training**<br>• 1 (with partner) = 44.4 % (*n* = 8)<br>• 2 (alone) = 55.6 % (*n* = 10)<br><br>**ARV_2: Recommendation of training**<br>*M* (*SD*) = 3.87 (1.38) (*n* = 55)<br>• $M_{G1\text{-}I}$ (*SD*) = 3.24 (1.35) (*n* = 17)<br>• $M_{G1\text{-}P}$ (*SD*) = 3.85 (1.39) (*n* = 20)<br>• $M_{G2\text{-}I}$ (*SD*) = 4.50 (1.15) (*n* = 18)<br><br>**Kruskal-Wallis test**<br>$H(2) = 7.845$, $p = .020$<br>Post-hoc comparison: $M_{G1\text{-}I} < M_{G2\text{-}I}$	**Ambiguous preference for partner participation**<br>>> wish for the same participant group<br><br>**Higher recommend-dation if participation without partner**	**INTERVENTION ACCEPTANCE: BENEFIT OF PARTNER (NON-) PARTICIPATION**<br>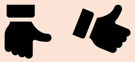	**Partner as beneficial:**<br>- Partner as motivating/ has motivated (G1-P)<br>- Partner as reminder/ has reminded (G1-P + G2-P)<br>- Participation was pleasant, joyful (G1-P + G2-P)<br>- Exchange of experience (G1-P)<br><br>**Partner as rather non-beneficial:**<br>- Different levels of motivation<br>- Permission to cheat	*„Doing it together always makes it easier. Achim: Exactly.// So no, we haven't slowed each other down, you rather motivate each other" (FG couples, 144).*<br><br>*„Even though he didn't participate […], it was important to him to remind me because he noticed it was good for me." (FG women, 114).*<br><br>*„I didn't need to be motivated by my wife. But it was pleasant to do these two hunger and satiety exercises together with her" (FG mixed, 456).*<br><br>*„When you do it together it's definitely more fun, obviously, than struggling with it by yourself. Then you can exchange experiences with one another: 'How was it like for you?'" (FG couples, 145)*<br><br>*„I have to say, at the beginning, I really had to convince him [partner] to participate at all" (FG women, 109).*<br><br>*„[laughs] if one cheats, the other one cheats too" (FG couples, 97).*

Note. Thumb symbols indicate positive and negative experiences/attitudes/evaluations; G1-I = Index person group 1, G1-P = partner group 1, G2-I = Index person group 2, G2-P = partner group 2, G3-I = Index person group 3, G3-P = partner group 3; IE = Intuitive Eating; IS = Interoceptive Sensitivity; BS=Body Scan, HU = hunger exercise, SA = satiety exercise; the names in the QUAL citations are pseudonyms

Synthesis Table according to the Pillar Integration Process (Johnson et al. 2017)

Reference

Johnson, R., Grove, A., und Clarke, A., Pillar Integration Process: A Joint Display Technique to Integrate Data in Mixed Methods Research. Journal of Mixed Methods Research, 2017. 13: p. 155868981774310.
